# Co-registration of eye movements and neuroimaging for studying contextual predictions in natural reading

**DOI:** 10.1080/23273798.2019.1616102

**Published:** 2019-05-16

**Authors:** Nicole A. Himmelstoss, Sarah Schuster, Florian Hutzler, Rosalyn Moran, Stefan Hawelka

**Affiliations:** a Centre for Cognitive Neuroscience, University of Salzburg, Salzburg, Austria; b Department of Neuroimaging, Institute of Psychiatry, Psychology & Neuroscience, King’s College London, London, UK

**Keywords:** Co-registration, eye movements, neuroimaging, fixation-related, natural reading, contextual predictions

## Abstract

Sixteen years ago, Sereno and Rayner (2003. Measuring word recognition in reading: eye movements and event-related potentials. *Trends in Cognitive Sciences*, *7*(11), 489–493) illustrated how “by means of review and comparison” eye movement (EM) and event-related potential (ERP) studies may advance our understanding of visual word recognition. Attempts to simultaneously record EMs and ERPs soon followed. Recently, this co-registration approach has also been transferred to fMRI and oscillatory EEG. With experimental settings close to natural reading, co-registration enables us to directly integrate insights from EM and neuroimaging studies. This should extend current experimental paradigms by moving the field towards studying sentence-level processing including effects of context and parafoveal preview. This article will introduce the basic principles and applications of co-registration and selectively review how this approach may shed light on one of the most controversially discussed issues in reading research, contextual predictions in online language processing.

## Introduction

Sixteen years ago, Sereno and Rayner (2003) pointed out how “by means of review and comparison” eye movements (EMs) and event-related potentials (ERPs) may advance our understanding of the “what”, “when” and “how” of visual word recognition in natural reading. Both methods contribute valuable insights on psycholinguistic processing in reading. EM studies provide sensitive measures of temporal and spatial progression of oculomotor control during reading that are indicative of processing effort at different psycholinguistic levels as well as attention allocation (Rayner, Sereno, Morris, Schmauder, & Clifton, 1989). ERPs, in contrast, provide reliable indications about the time-course of neural activity associated with cognitive mechanisms – however lack information on corresponding EM behaviour. Although findings from ERP and EM studies are therefore complementary, their comparison in terms of temporal processing are somewhat incongruous. That is, the time-course of visual word recognition reported in traditional ERP studies exceeds normal fixation durations during natural reading, impeding its integration into the processing timeline as derived from EM studies. Supposedly, this divergence is due to different experimental protocols: EM studies allow participants to read whole sentences or paragraphs at their own pace, whereas ERP studies prevent normal reading behaviour by imposing rapid serial visual presentation (RSVP) of isolated words. Undoubtedly, ERPs have proven to be a valuable method to study visual word recognition (see Kutas & Federmeier, 2011 for a review). However, breaking behaviour and experience down into a sequence of externally triggered events comes with limitations, questioning the suitability of RSVP with regard to studying online visual language processing. RSVP imposes three sorts of limitations:

1. Events are presented at a fixed pace, usually with presentation durations of 500–1000 ms per word, that is a multiple of the duration of a typical eye fixation during natural reading (∼200–250 ms; Kliegl, Dambacher, Dimigen, & Sommer, 2014; Rayner, 1998, 2009). This might alter, for example by artificially prolonging, the time-course of visual word recognition (Dambacher et al., 2012). As a consequence, reading-related brain regions might be recruited beyond the intrinsic level necessary for visual word recognition during natural reading and thus show an increase in neural activation beyond naturalistic settings (Schuster, Hawelka, Richlan, Ludersdorfer, & Hutzler, 2015).
2. EMs typical for natural reading such as word skippings or regressive saccades, are prevented. Disrupting dynamics of syntactic parsing and semantic processing as well as impairing ambiguity resolution (e.g. when encountering unexpected linguistic input) are assumed to result not only in different processing demands, but also reading strategies and, as a result, affect reading comprehension (Kliegl, Dambacher, Dimigen, Jacobs, & Sommer, 2012; Metzner, von der Malsburg, Vasishth, & Rösler, 2017; Rayner, 1998; Schotter, Tran, & Rayner, 2014).
3. Parafoveal preview, that is pre-processing of an upcoming word, is prohibited. Critically, parafoveal pre-processing is assumed to facilitate word recognition by about 30–60 ms once a word is fixated (Rayner, 1975). Preventing parafoveal preview thus most likely causes differences in the time-course of visual word processing (Hutzler et al., 2013; Kliegl et al., 2012, 2014; Kornrumpf, Niefind, Sommer, & Dimigen, 2016).

Investigating visual language processing therefore requires a method that is not only able to track the rate at which reading proceeds, but also to determine the informational role of EM behaviour and corresponding neural correlates of visual word recognition. In terms of an experimental setting close to natural reading, co-registration of EMs and neuroimaging meets these requirements.

## Co-registration: investigating visual word recognition during natural reading

**Figure 1.**
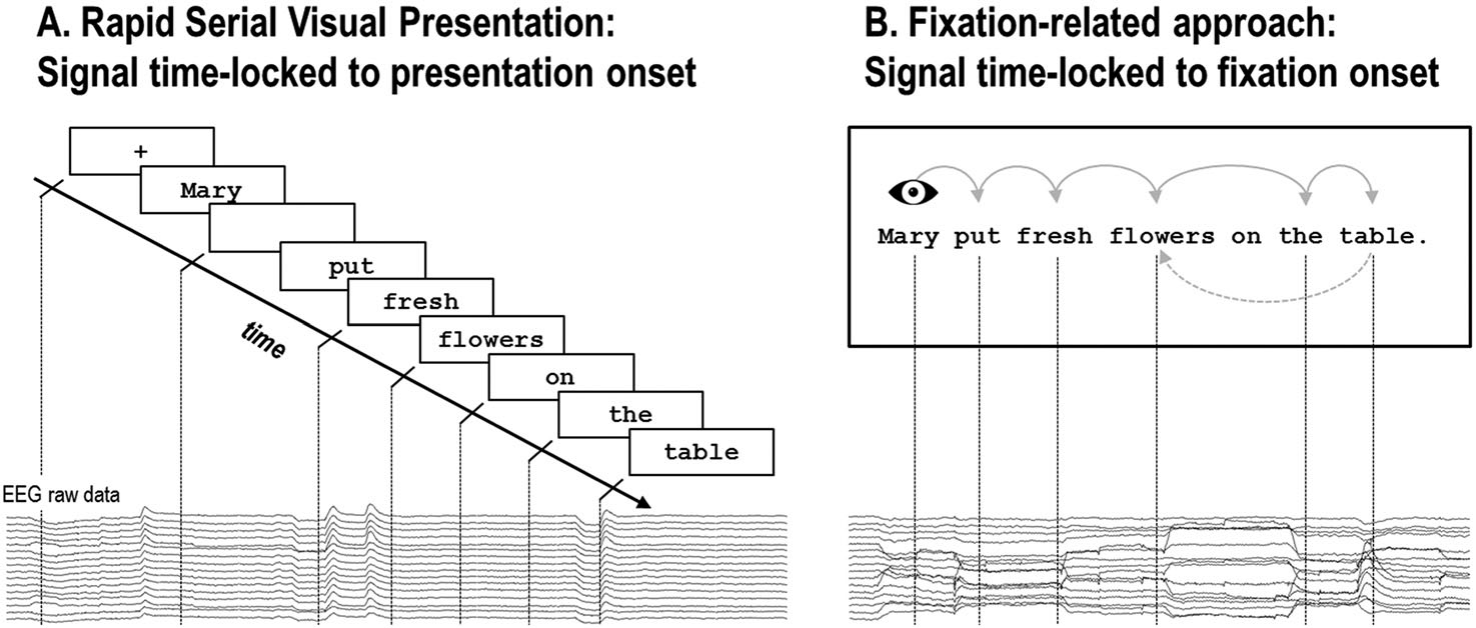
Schematic illustration of (A) the RSVP and (B) the fixation-related approach. In RSVP, individual words are presented one-by-one at the centre of a screen, usually preceded by a fixation-cross and intermitted by a blank screen (for illustrative purposes only shown for the first word). Analysis of the event-related signal proceeds time-locked to the externally triggered onset of a particular word. By contrast, in the fixation-related approach words are presented simultaneously, typically in a single line. Participants read at their own pace while their EMs are recorded. This approach allows participants (i) to endogenously allocate their attention and execute saccades, (ii) to parafoveally pre-process the upcoming word(s), (iii) to skip words and (iv) to reinspect formerly encountered words by means of regressive saccades. The EEG signal then is analysed time-locked to the point in time of, commonly, the first fixation on a word.

Co-registration is based on the simultaneous application of eye tracking and neuroimaging techniques (e.g. EEG, fMRI), allowing for joint analysis of synchronised EM and neuroimaging time series. By this means, instead of analysing event-related brain activity time-locked to an externally triggered stimulus onset as with RSVP (e.g. ERPs), co-registration enables the investigation of brain activity time-locked to the onset of a fixation on, for example, a target in visual search paradigms (e.g. Brouwer, Reuderink, Vincent, van Gerven, & van Erp, 2013; Devillez, Guyader, & Guérin-Dugué, 2015; Finke, Essig, Marchioro, & Ritter, 2016; Kamienkowski, Ison, Quiroga, & Sigman, 2012; Kaunitz et al., 2014; Körner et al., 2014; Wenzel, Golenia, & Blankertz, 2016), a spatial location in picture or scene viewing (Fischer, Graupner, Velichkovsky, & Pannasch, 2013; Henderson & Choi, 2015; Marsman, Renken, Velichkovsky, Hooymans, & Cornelissen, 2012; Nikolaev, Jurica, Nakatani, Plomp, & van Leeuwen, 2013; Nikolaev, Nakatani, Plomp, Jurica, & van Leeuwen, 2011; Ossandón, Helo, Montefusco-Siegmund, & Maldonado, 2010), or, in case of reading, a particular word during self-paced reading of multi-word stimuli, such as word lists, sentences or paragraphs (see Figure 1).

### Fixation-related approaches used in reading research

The first approach used in the study of reading was – as an equivalent to ERPs – *fixation-related potentials (FRPs)*. Initially it has been demonstrated that FRPs yield similar results to conventional ERPs obtained during RSVP (e.g. Dimigen, Sommer, Hohlfeld, Jacobs, & Kliegl, 2011; Hutzler et al., 2007; Kornrumpf et al., 2016; Kretzschmar, Bornkessel-Schlesewsky, & Schlesewsky, 2009; Metzner et al., 2017; Metzner, von der Malsburg, Vasishth, & Rösler, 2015). However, findings also indicate that electro-physiological correlates of cognitive processes might occur earlier in self-paced reading compared to RSVP and, moreover, are related to EM measures indicative of processing difficulty (Dimigen et al., 2011). FRPs have also successfully been applied to examine the impact of parafoveal pre-processing on visual word recognition (Baccino & Manuta, 2005; Degno et al., 2018; Dimigen, Kliegl, & Sommer, 2012; Dimigen, Sommer, Dambacher, & Kliegl, 2008; Kornrumpf et al., 2016; Simola, Holmqvist, & Lindgren, 2009) and, more recently, to investigate effects of visual processing load on orthographic processing and its relation to reading proficiency (Weiss, Knakker, & Vidnyánszky, 2016).

Although FRPs are a reliable and valid measure for the timing of cognitive processes, due to the low spatial resolution of EEG, their suitability for localising brain regions ascribed with certain aspects of language processing is limited (Jobard, Crivello, & Tzourio-Mazoyer, 2003; Price, 2012; Taylor, Rastle, & Davis, 2013). Consequently, co-registration has also been transferred to functional magnetic resonance imaging (fMRI), named *fixation-related fMRI*. Here, similar to FRPs, instead of using external triggers, the fixation-onset during online reading serves as a marker for modelling haemodynamic brain responses (Marsman et al., 2012; Richlan et al., 2014). The combined recording of EMs and fMRI has been used to investigate the neural underpinnings of natural EM behaviour during reading (Choi, Desai, & Henderson, 2014; Henderson, Choi, Luke, & Desai, 2015; Henderson, Choi, Luke, & Schmidt, 2018) and the generalisability of effects observed during isolated visual word recognition to natural reading (Bonhage, Mueller, Friederici, & Fiebach, 2015; Desai, Choi, Lai, & Henderson, 2016; Henderson, Choi, Lowder, & Ferreira, 2016; Schuster, Hawelka, Hutzler, Kronbichler, & Richlan, 2016). Together, these findings provide a first proof-of-concept that fixation-related fMRI may pave the way not only for identifying brain areas engaging in natural reading in a spatially sensitive manner, but also to further our understanding of whether reading-related activation patterns within these regions can be attributed to specific representational levels (e.g. phonology, orthography, semantics; Carreiras, Armstrong, Perea, & Frost, 2014).

A rather new approach is *fixation-related oscillatory EEG*, which follows the same methodological principles as the above described fixation-related approaches, but with neuronal oscillations. Neuronal oscillations are assumed to reflect rhythmic changes of high and low levels of cortical excitability caused by fluctuating synaptic inputs and consequent firing rates of neuronal assemblies within various frequencies at multiple spatial scales. Moreover, they are supposedly distinctive with regard to their perceptual and cognitive functionality (Bassett, Meyer-Lindenberg, Achard, Duke, & Bullmore, 2006; Bressler, 1995; Buzsáki & Draguhn, 2004; Cohen, 2017; Hipp, Engel, & Siegel, 2011; Klimesch, Sauseng, & Hanslmayr, 2007). So far, fixation-related oscillations have been used to investigate online semantic and syntactic sentence-processing (Metzner et al., 2015; Vignali, Himmelstoss, Hawelka, Richlan, & Hutzler, 2016) as well as attention allocation during word-list reading (Kornrumpf, Dimigen, & Sommer, 2017). Although findings regarding fixation-related oscillations up to now are scarce, we will try to argue that this approach holds great promise in investigating the neural mechanisms underlying natural reading (see Section “New perspectives: the role of neuronal oscillations during natural reading”).

### Challenges

Co-registration of EMs and neuroimaging comes with technical and methodological challenges, particularly in the case of EEG. These aspects are beyond the scope of this article and therefore will only briefly be outlined. The interested reader may consult the below referenced work for an in-depth discussion:

1. *Trigger synchronisation:* The foremost requirement for co-registration is a proper synchronisation of the recording devices, that is, the eye tracking system and EEG or fMRI. This synchronisation has to be guaranteed throughout the whole recording (preferably by continuous triggering), since minimal drifts in the clocks of the devices can amount to substantial deviations (in the order of milliseconds). One way to ensure that triggers to the EM and the EEG datastream are synchronous, is to use split trigger pulses. A possibility to assess potential time delays of triggers is using a photosensitive diode to measure the timing of stimulus presentation, the detection of EMs by the eye tracker and the arrival of the triggers from the EEG (or fMRI; e.g. Richlan et al., 2013).
2. *Correction of ocular artefacts:* More difficult is the proper correction of saccadic EMs preceding the time window of analysis and causing non-neural artefacts such as rotation of the corneo-retinal dipole or oculomotor muscles. Additionally, neural artefacts including presaccadic potentials related to motor preparation or perceptual suppression potentially may exceed the neural activity of interest (see e.g. Baccino, 2011; Dimigen et al., 2011; Henderson, Luke, Schmidt, & Richards, 2013; Plöchl, Ossandón, & König, 2012 for comprehensive overviews). Regression as well as independent component analysis (ICA) based approaches were proposed to correct for these artefacts (see Dimigen et al., 2011; Henderson et al., 2013; Plöchl et al., 2012; please also see the EYE-EEG toolbox for simultaneous eye tracking and EEG analysis; Dimigen et al., 2011), including recommendations on how to optimise ICA procedures for free viewing experiments, such as natural sentence reading (Dimigen, 2018).
3. *Deconvolution of overlapping signals:* Probably most challenging are overlapping brain responses caused by rapidly succeeding fixations. During reading, typical fixations last (on average) 200–250 ms and are thus much shorter than time windows commonly used for EEG analysis. For example, when analysing the N400, at the time this component arises, the reader – more often than not – is no longer fixating the word of interest. As a consequence, components evoked by the subsequent word coincide with the ongoing processing of the previous word, resulting in overlapping potentials. The issue of temporal overlap can – at least to some extent – be circumvented by trying to keep overlapping components constant, for example by using identical sentence frames counterbalanced across participants. Such an approach, however, is not suitable for all research questions. Recently, Ehinger and Dimigen (2018) offered a solution for this issue by means of deconvolution which overcomes limitations of previous approaches (such as, e.g. the ADJAR method; Woldorff, 1993). For the event-related analysis of fMRI temporal overlaps are an inherent challenge due to the slow dynamics of the BOLD signal, but are successfully dealt with because of the (fairly) linear additivity of the BOLD signal (Dale & Buckner, 1997).

Besides these challenges, in our view, the foremost advantages of co-registration with respect to investigating visual word recognition are as follows:

1. Participants can process words at their own pace. Therefore inherent temporal dynamics of visual word recognition during reading are maintained (e.g. Dimigen et al., 2011).
2. Co-registration allows us to assess effects of EM control such as word skippings (Schuster et al., 2016) or regressive EMs (Metzner et al., 2017) and thus holds the potential to contribute to the further development of models of EM control during reading.
3. Co-registration allows the reader to parafoveally pre-process the upcoming word(s), providing insights into the nature of information being extracted from parafoveal vision (e.g. Dimigen et al., 2012; Niefind & Dimigen, 2016).
4. EMs can serve as an externally observable indicator for the engagement of the participant, rendering explicit tasks (e.g. lexical decision) – which may alter brain responses – unnecessary (e.g. Reilly, 2014; Schuster et al., 2015).

In the following we will sketch existing and potential research endeavours which could be further advanced by means of co-registration. We particularly focus on contextual predictions in sentence comprehension and objectives to determine the time-course of top-down and bottom-up mechanisms: (i) disentangling context-based integration from pre-activation and (ii) investigating additive and interactive effects of supposedly bottom-up and top-down determinants of visual word recognition. We will compare findings from ERPs and FRPs, but will also incorporate evidence from EM studies, especially on the role of parafoveal pre-processing in visual word recognition. Finally, we will elaborate on new perspectives in reading research with a special emphasis on fixation-related oscillatory EEG and neural network dynamics.

## The “when and how” of contextual predictions in visual word recognition

Natural reading proceeds at rates of up to 350 words per minute indicating fast and dynamic orchestration of perceptual, attentional and cognitive processing. Though there is broad consensus about which processes engage in reading, little is known about how information is organised to enable ongoing transition from perception to comprehension. An important debate in this respect is whether word recognition commences upon bottom-up lexical processing of a word, subsequently followed by post-lexical extraction of its meaning, or rather top-down modulated lexical processing based on pre-activated word meaning or *contextual predictions*.^1^ Critically, the question thus is *not if, but when and how* context affects visual word recognition, more precisely, at which stage of processing (lexical vs. post-lexical) bottom-up and top-down mechanisms become effective and in what way, if so, they interact with each other.

In EM research, the effect of sentential context upon word recognition is typically assessed by measuring the impact of *word predictability* ^2^, a factor known to affect the speed of visual word recognition (for reviews see Rayner, 1998, 2009; Staub, 2015). Word predictability is thought to serve as a proxy of top-down expectations (e.g. Kliegl et al., 2014) as evidenced by shorter fixation durations and higher skipping probabilities for contextually predictable compared to unpredictable words (e.g. Balota, Pollatsek, & Rayner, 1985; Ehrlich & Rayner, 1981; Hawelka, Schuster, Gagl, & Hutzler, 2015; Kliegl, Grabner, Rolfs, & Engbert, 2004; Kliegl, Nuthmann, & Engbert, 2006; Rayner, Binder, Ashby, & Pollatsek, 2001; Rayner & Well, 1996). A question that has long been a subject of controversy, both in EM and neuroimaging research on reading, is whether facilitating effects of predictability are due to rapid *context-based integration* of encountered words (“integration view”) or *context-based pre-activation* of words and/or word features before they are encountered (“prediction view”). At the centre of the debate is one of the most well-documented event-related components in visual word recognition: the N400 – a negative-going deflection in the time-locked EEG signal (depending on the electrode site relative to the recording reference) with a peak amplitude at around 400 ms post-stimulus (Kutas & Federmeier, 2011).

### The semantic N400: context-based integration or pre-activation?

From the perspective of the “integration view”, visual word recognition is primarily driven by bottom-up lexical processing, initiating post-lexical retrieval of semantic representations and its integration into prior context. The “prediction view”, on the contrary, assumes that visual word recognition operates via top-down pre-activation of word meaning, “gating” bottom-up *lexical access* ^3^ (DeLong, Troyer, & Kutas, 2014; Kutas, DeLong, & Smith, 2011; Kutas & Federmeier, 2011; Staub, 2015; Van Petten & Luka, 2012). There is extensive evidence that the N400 serves as an index for context-dependent processing demands and is modulated by various experimental manipulations including semantic congruency, plausibility, semantic relatedness or cloze probability. However, despite decades of research following the first report on this component by Kutas and Hillyard (1980), there is still no consensus whether reduced N400 amplitudes in response to contextually predictable target words reflect the ease with which a word is integrated into prior context or (probabilistic) pre-activation of plausible continuations (for an overview see Table 1; for reviews see Kuperberg, 2016; Kuperberg, Kreher, & Ditman, 2010; Kutas & Federmeier, 2000; Kutas & Federmeier, 2011; Lau, Phillips, & Poeppel, 2008; Swaab, Ledoux, Camblin, & Boudewyn, 2012). The debate has even been broadened by questioning a clear distinction between the two theoretical stances (Nieuwland et al., 2018a) by arguing in favour of a “multiple-process” account (e.g. Baggio, 2012; Baggio & Hagoort, 2011).

**Table 1.** Overview of studies investigating contextual effects on the N400.

Authors	Manipulation	Integration	Pre-activation	Multi-process
Kutas and Hillyard (1984)	Cloze probability, Semantic relatedness		X	
Federmeier and Kutas (1999)	Cloze probability, Semantic relatedness		X	
van Berkum, Hagoort, & Brown, (1999)	Semantic congruency	X		
Kuperberg, Sitnikova, Caplan, and Holcomb (2003)	Thematic role animacy violation, non-thematic role pragmatic violation	X		
Hagoort, Hald, Bastiaansen, and Petersson (2004)	World knowledge violation, semantic violation	X		
DeLong, Urbach, and Kutas (2005)^a^	Cloze probability		X	
Dambacher, Kliegl, Hofmann, and Jacobs (2006)	Cloze probability		X	
Thornhill and Van Petten (2012)	Cloze probability, Semantic relatedness		X	
Wlotko and Federmeier (2012)	Cloze probability			X
Martin et al. (2013)^a^	Cloze probability		X	
Ito et al. (2017a)^a^	Cloze probability	X		
Nieuwland et al. (2018a)	Cloze probability, plausibility			X
Nieuwland et al. (2018b)^a^	Cloze probability			X
Szewczyk and Schriefers (2018)	Semantic congruency, repetition priming		X	

^a^Evidence stemming from pre-target analyses.

Note: Please note that this is not an exhaustive overview. Furthermore, it must be emphasised that some studies declared as being more in favour of a pre-activation account might not qualify as evidence for the “strong” version of the prediction view (see Van Petten & Luka, 2012).

Investigating pre-target intervals has the potential to contribute to this discussion. By presenting sentences like “The day was breezy so the boy went outside to fly a/an kite/airplane” DeLong et al. (2005) not only replicated previously reported N400 effects in response to unpredicted nouns (“airplane” in the above example), but could also demonstrate that this effect was already present on the preceding article (“an” in the above example). According to the authors, this can only reasonably be interpreted in terms of probabilistic pre-activation of the phonological word form of the upcoming noun, since the article itself does not impose differences in integration difficulty. Similar findings have been reported by Martin et al. (2013) in native compared to non-native readers. Having said that, attempts to replicate these findings, so far, have not been successful (Ito, Martin, & Nieuwland, 2017a; Nieuwland et al., 2018b), which, according to Nieuwland and colleagues, might not only question a “strong prediction view”, but also fuel a more general discussion on the significance of predictions for language comprehension (Huettig, 2015; Huettig & Mani, 2016). While this debate is yet to be concluded (DeLong, Urbach, & Kutas, 2017; Ito, Martin, & Nieuwland, 2017b; Yan, Kuperberg, & Jaeger, 2017), investigating alterations of brain signals as a function of the “expectedness” of potential upcoming words within the pre-target time interval remains a promising approach to address the issue of word pre-activation and therefore has also been subject to FRP studies.

### Evidence from FRPs

An FRP study explicitly addressing context-based integration versus pre-activation was done by Kretzschmar et al. (2009). The authors made use of antonym-constructions (e.g. “The opposite of black is … ”) ending either with the predicted antonym (“white”), an unpredicted but semantically related word (“yellow”) or an unpredicted and semantically unrelated word (“nice”). In line with previous findings, Kretzschmar and colleagues found higher N400 amplitudes and prolonged first fixation durations for unpredicted target words (“yellow”, “nice” > “white”). Interestingly, analysis based on the last fixation prior to the target word also revealed an N400 effect, yet only in the semantically unrelated condition (“nice” > “white”, “yellow”). While, according to the authors, the target-elicited N400 effect can be explained by both, the integration and the prediction view, the “pre-target” effect indicates (broad) lexical pre-activation of the expected antonym and semantically related words. Such lexical pre-activation however differs from those exposed by studies of DeLong et al. (2005) and Martin et al. (2013) in that it has not been induced by a mismatch between the pre-target word and pre-activated features of the expected continuation, but by pre-activated features and the *parafoveally pre-processed* continuation (for similar findings see Metzner et al. (2015) who directly compared ERPs and FRPs with a world knowledge paradigm).

Critically, however, the onset of the purportedly parafoveally induced N400 effect (∼250 ms) reported by Kretzschmar et al. (2009) exceeded the average duration of the last fixation in the pre-target region (∼186 ms) and thus must have coincided with fixations on the subsequent target words. Hence, it is possible that the effect was not induced by parafoveal pre-processing, but is actually an effect induced upon fixating the target word which overlaps with the preceding “pre-target” FRP (see section “Challenges – Deconvolution of overlapping signals”). Indeed, visual inspection reveals a close similarity between the gradation of the “parafoveal” N400 effect and an early component elicited by the target word. However, this effect could still be driven or at least influenced by word pre-activation and/or parafoveal pre-processing. To illustrate how two processes may contribute to this effect, let us consider how top-down pre-activation of the upcoming word may not initially interact with visual bottom-up information provided by the parafoveal word as long as the reader fixates the preceding word. Put differently, there may be no parafoveal-on-foveal influence since the two streams of information coincide only upon fixating a word, but then this may happen almost instantaneously (possibly as early as 80 ms post-fixation; Dimigen et al., 2012).

 Furthermore, there is some evidence indicating a dissociation of (first) fixation durations and fixation-related N400 amplitudes as evidenced by a corpus analysis conducted by Dimigen et al. (2011). Based on 144 sentences – comprising words with varying cloze probability (Potsdam Sentence Corpus; Kliegl et al., 2004) – the authors observed shorter first fixation and gaze duration for high than low-predictable words and robust N400 predictability effects, peaking at around 384 ms after fixation-onset. However, when the N400 reached its peak, in 96% of the cases the initial fixation has already been terminated. It was further noted that N400 amplitudes were more closely related to gaze duration than to first fixation duration. Yet given a mean gaze duration of 278 ms, the authors questioned the possibility that this behavioural effect was indeed driven by the same neural generators as the N400 effect. Still, this finding seems to be in accordance with the notion that the N400 exceeds the time-course given for lexical processing with an upper bound between 200 and 250 ms during natural reading and therefore rather reflects post-lexical processing (Rayner, 1998; Sereno & Rayner, 2003; Sereno, Rayner, & Posner, 1998). In general, one might argue that this finding is in line with the “integration view”, that is sequential processing of bottom-up lexical and top-down post-lexical information, with the latter being reflected in the N400.

Having said this, predictability effects have also been observed *prior* to the presumed temporal constraint for post-lexical processing. For instance, an ERP study by Dambacher, Rolfs, Göllner, Kliegl, and Jacobs (2009) revealed a difference between predictable and unpredictable target words as early as around 90 ms post-stimulus. In line with Dambacher et al. (2009), Lewis, Schoffelen, Hoffmann, Bastiaansen, and Schriefers (2017) reported effects of semantically coherent compared to incoherent discourses already between 80 and 200 ms relative to word onset. Importantly, these findings seem to correspond to EM research, estimating the earliest effect of word predictability on fixation duration at approximately 140 ms (Sheridan & Reingold, 2012). If this holds true, predictability effects however must temporally coincide with effects of *word frequency* ^4^ – arguably a bottom-up determinant of lexical access (Inhoff, 1984; Kliegl et al., 2004; but see Chen, Davis, Pulvermüller, & Hauk, 2015; Strijkers, Bertrand, & Grainger, 2015). As for word predictability, EM studies consistently demonstrated facilitating effects of word frequency on visual word recognition as indicated by shorter fixation durations and higher skipping probabilities for frequent than infrequent words (Henderson & Ferreira, 1993; Hyönä & Olson, 1995; Inhoff & Rayner, 1986; Kliegl et al., 2004, 2006; Rayner & Duffy, 1986; Rayner, Sereno, & Raney, 1996; Schilling, Rayner, & Chumbley, 1998; Slattery, Pollatsek, & Rayner, 2007). Critically, Reingold, Reichle, Glaholt, and Sheridan (2012) estimated that word frequency exerts its influence on fixation duration as early as 145 ms post-fixation, that is within the same time window as word predictability. Likewise, several ERP studies reported frequency effects emerging within the first 200 ms post-stimulus (e.g. Hauk, Davis, Ford, Pulvermuller, & Marslen-Wilson, 2006; Hauk & Pulvermüller, 2004; Reichle, Tokowicz, Liu, & Perfetti, 2011; Sereno et al., 1998; but see Laszlo & Federmeier, 2014 for a critical discussion). It must be noted, that there are also reports on frequency effects within the N400 time window, which, however, mainly seem to arise from interactions with other factors including word repetition and length (e.g. King & Kutas, 1998; Rugg, 1990). Of particular interest in this respect are word position effects, showing a gradual decrease in N400 amplitudes with increasing word position (Van Petten, 1993; Van Petten & Kutas, 1990, 1991). Critically, when accounting for word predictability and the interaction between word predictability and frequency, Dambacher et al. (2006) could demonstrate that the effect of word position on the N400 gets assimilated, which, according to the authors, substantiates the notion that word position can be considered as a proxy of contextual constraint (but see Schuster, Hawelka, Himmelstoss, Richlan, & Hutzler [in press] for dissociable effects of word position and predictability). More importantly, however, this study revealed an interaction between word predictability and frequency, indicating that when context is given, the impact of lexical frequency on the N400 seems to be attenuated.

Taken together, findings with respect to the question at which processing stage contextual predictions become effective are still inconclusive. Admittedly, most of the neuroimaging studies so far reported late effects of word predictability (i.e. within the N400 time window), whereas word frequency seems to exert its effect comparatively early (i.e. within the P200 time window), suggesting a primacy of lexical processing. Critically, however, there is also some evidence for early context-dependent ERP effects. Moreover, EM studies demonstrated that both, word predictability *and* frequency become effective within the time frame of lexical access (e.g. Sereno & Rayner, 2003), while FRP studies indicate that effects of word predictability on fixation duration do not align with those on N400 amplitudes. An alternative approach which may inform the debate on contextual influences on word recognition originates from EM research: investigating whether early effects of word frequency and predictability interact or are merely additive (e.g. Hand, Miellet, O’Donnell, & Sereno, 2010). Applying this reasoning to F/ERPs, in consequence, requires a focus on early rather than late components.

### Before the N400: additive or interactive effects?

**Figure 2.**
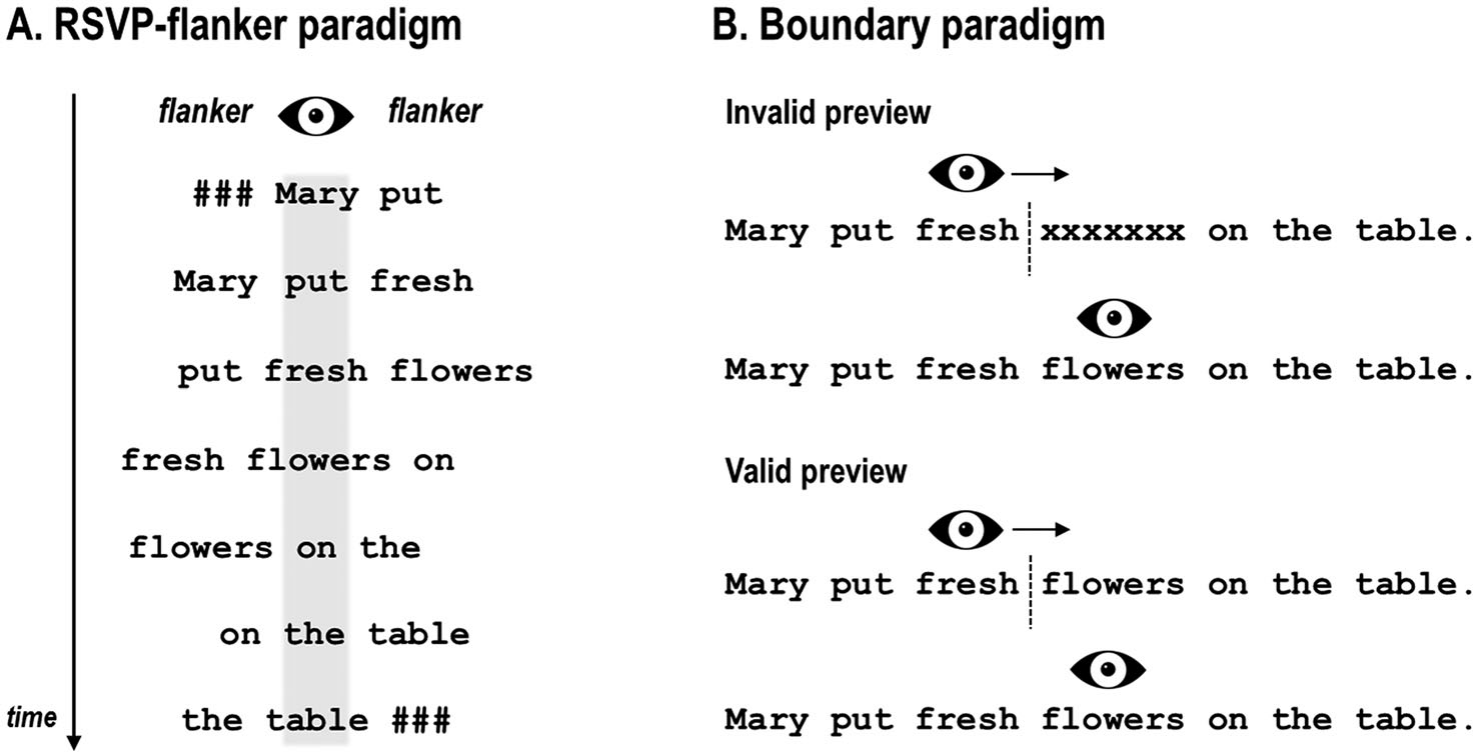
Schematic illustration how parafoveal pre-processing is typically investigated in (A) ERP studies and (B) EM studies. In ERP studies using the RSVP-with-flankers paradigm participants are instructed to maintain central fixation to avoid EM artefacts. The fixated word is flanked by, e.g. the (previous and) next word in a sentence. After a certain constant duration, the fixated word is replaced by the parafoveal word which, in turn, is replaced by the consecutive word of the sentence, *asf*. In EM studies, parafoveal pre-processing is typically investigated with the boundary paradigm (Rayner, 1975) in which the preview of a parafoveal target word is experimentally manipulated until a pre-target boundary is crossed. Often, the manipulation is masking the target word, e.g. with a string of “X”s or different letters of equal length (see also Hutzler et al., 2013; Kliegl, Hohenstein, Yan, & McDonald, 2013; Marx, Hawelka, Schuster, & Hutzler, 2015 for cautionary notes on using parafoveal masks in the boundary paradigm). A recent study comparing the flanker and the boundary paradigm revealed that the preview effect is substantially larger in the boundary paradigm, indicating that passive reading (i.e. without saccades) in the flanker paradigm does not assimilate natural (i.e. active) reading with saccades (Kornrumpf et al., 2016). Recent FRP studies on parafoveal pre-processing during natural reading which made use of the boundary paradigm reported early effects of valid previews over occipitotemporal electrodes (e.g. Dimigen et al., 2012; Niefind & Dimigen, 2016).

It has been proposed in the EM literature, that an additive effect of word frequency and predictability would point to a primacy of bottom-up processing with contextual effects emerging post-lexically, whereas an interaction of these variables would be indicative of an early effect of context on lexical processing (Hand et al., 2010; Sereno, Hand, Shahid, Yao, & O’Donnell, 2018). Interestingly, EM studies investigating word frequency and predictability conjointly, mainly reported additive effects, that is, both variables contribute to fixation duration and skipping probability, yet independent of each other (e.g. Altarriba, Kroll, Sholl, & Rayner, 1996; Ashby, Rayner, & Clifton, 2005; Fitzsimmons & Drieghe, 2013; Kliegl et al., 2004, 2006; Lavigne, Vitu, & d'Ydewalle, 2000; Miellet, Sparrow, & Sereno, 2007; Rayner, Ashby, Pollatsek, & Reichle, 2004; Rayner et al., 2001; see Staub, 2015 for a review). A recent study by Sereno et al. (2018), however, puts these findings into perspective. In contrast to previous studies, Sereno and colleagues did not only manipulate word predictability and frequency of target words, but also the availability of their parafoveal preview (valid vs. invalid) by means of the *boundary paradigm* (see Figure 2). Importantly, while both preview conditions led to independent frequency and predictability effects on fixation times, only in the valid preview condition an interaction could be observed which has arised from a diminished frequency effect for high compared to medium and unpredictable words. Indeed, this finding provides reliable evidence for the impact of parafoveally pre-processed contextual information on early lexical processing during natural reading. Critically, one might argue, that a statistical interaction of word frequency and predictability in behavioural data, such as fixation durations, does not necessarily imply simultaneous processing, since fixation duration may only reflect the “endpoint” of processing stages which in itself still can be sequential. Respective conclusions, however, could be drawn from neural measures, such as, for example ERPs.

In contrast to the majority of EM studies, there is some evidence for early interactive effects in ERPs (Lee, Liu, & Tsai, 2012; Sereno, Brewer, & O’Donnell, 2003; Penolazzi, Hauk, & Pulvermüller, 2007). Of particular interest in this respect is a finding indicating that the emergence of interactive effects in RSVP studies is modulated by stimulus-onset-asynchrony (SOA), that is, the time between stimulus onsets (Harley, 2014). Here, Dambacher et al. (2012) could demonstrate that early interactive effects only emerge in case of short SOAs (280 ms), suggesting context-based enhancement of early lexical processing when RSVP approximates the rate at which natural reading proceeds. In general, this finding corroborates the notion that imposing a predefined time-window for information processing by use of RSVP might bias the engagement of cognitive processes (see also Brothers, Swaab, & Traxler, 2015; Wlotko & Federmeier, 2015) and therefore alter the time-course of visual word recognition. Having said that, even in case of short SOAs – as has also been pointed out by Dambacher et al. (2012) – the question remains open whether inferences based on RSVP findings can be transferred to natural reading.

### Evidence from FRPs

To our knowledge, only one FRP study explicitly investigated potential interactions between word frequency and predictability, by presenting sentences with high and low-constraining target words, which were either high or low-frequent (Kretzschmar, Schlesewsky, & Staub, 2015). Analysis of the target words not only revealed reduced N400, but also enhanced P200 amplitudes for predictable compared to unpredictable words. However, no effect of word frequency or an interaction of frequency and predictability could be observed – neither in early nor late components. On the pre-target level, FRPs showed no impact of predictability or frequency of the upcoming target word (see Degno et al., 2018 for a similar finding). Behaviourally, while fixation durations on the target words were influenced by frequency and predictability in an additive fashion, analysis of the pre-target words revealed an interactive effect with longer fixation durations for unpredictable, high frequent words. Critically, as has also been pointed out by the authors, words preceding the target word were not matched across conditions with regard to word length and frequency. Thus, the observed interaction might have resulted from differences in the extent of parafoveal pre-processing due to varying *saccade launch site* ^5^ – a factor that has recently been shown to give rise to interactive effects in EMs (Hand et al., 2010; but see Slattery, Staub, & Rayner, 2012) Still, Kretzschmar and colleagues concluded, that when context is given, in contrast to EMs, the N400 seems to be insensitive to word frequency. Thus, word frequency might not carry additional information important for verifying top-down predictions.

To conclude, evidence in favour of early interactive effects in visual word recognition is scarce – particularly in EM studies. Still, initial findings on an early interplay of top-down and bottom-up determinants of visual word recognition in ERPs when approximating natural reading rates (Dambacher et al., 2012) and in EM behaviour when taking parafoveal preview into account (Sereno et al., 2018), clearly needs further investigation. Furthermore, to reliably assess whether potential early interactive effects in neural responses correspond to those in EM behaviour evidently necessitates the alignment of both measures. Co-registration of EMs and EEG may achieve a convergence of these findings. However, up to now only one FRP study explicitly addressed interactive effects between word predictability and frequency, yet without controlling for parafoveal pre-processing, limiting a meaningful interpretation. Thus, FRP studies emphasising early interactive effects while considering parafoveal preview as a potentially modulating factor might be a promising future endeavour to further specify the time-course of visual word recognition during natural reading and, in turn, contribute to the discussion on the significance of contextual predictions for online language processing.

## New perspectives: the role of neuronal oscillations during natural reading

In the previous sections we have sketched how FRPs may contribute to the issue of contextual predictions during natural reading by investigating the time-course of top-down and bottom-up processing inferred from evoked components. However, referring to these mechanisms also implies that reading engages an orchestrated interaction between brain regions acting at different levels of input processing (e.g. Carreiras et al., 2014) and, as a consequence, at different *hierarchically organised cortical levels*. Top-down and bottom-up, in this respect, can therefore also be referred to as feedback and feedforward information transmission (but see Rauss & Pourtois, 2013 for an overview of alternative definitions) between higher cortical levels – involved in syntactic and semantic information processing – and lower cortical levels – associated with visual-orthographic and lexical information processing. The question thus arises as to *when and how* information is integrated and transmitted within and between these levels. Investigating neuronal oscillations (commonly inferred from standard time–frequency analysis; Cohen, 2017; but see Haller et al., 2018), which are assumed to be indicative of intra- and inter-areal communication within cortical networks (e.g. Bressler & Richter, 2015; Engel, Fries, & Singer, 2001; Hipp et al., 2011; Varela, Lachaux, Rodriguez, & Martinerie, 2001; von Stein, Chiang, & König, 2000), holds great promise to contribute to this question. Although findings with respect to language processing vary widely depending on the experimental manipulation and methodological approach (for reviews see Bastiaansen & Hagoort, 2006; Lewis, Wang, & Bastiaansen, 2015; Meyer, 2018), investigating predictive processing during reading by means of oscillatory brain activity is a growing research area. Of particular interest in this respect is a recent theoretical framework for sentence-level language comprehension trying to link predictive coding theories with oscillatory network dynamics gating hierarchical information transmission in the language network (Lewis & Bastiaansen, 2015).

### Predictive coding in neuronal oscillations during reading

In brief, predictive coding posits that the brain continuously performs context-sensitive perceptual inference to optimise precision of sensory predictions and, as a result, reduce uncertainty about upcoming events (Clark, 2013; Friston, 2005, 2009, 2010; Rao & Ballard, 1999). This theory prescribes a hierarchically organised cortical architecture with reciprocal, yet functionally asymmetric backward (i.e. top-down) and forward (i.e. bottom-up) connections between higher and lower cortical levels. Predictions are thought to be generated in higher levels, descending to the next lower level, where they are compared with sensory inputs resulting in a so-called prediction error (PE), that is, the difference between expected and actual incoming information. The error signal subsequently propagates up the hierarchy to the next higher level to adjust the current prediction by updating the generative model and inferred likeliest causes. The revision of the predictive model is ongoing, aiming at minimising PEs at all levels within the hierarchy. Importantly, in some models of predictive coding, the impact of PEs on model updating varies as a function of weighting predictions according to their environmental evidence, that is, their precision (Adams, Stephan, Brown, Frith, & Friston, 2013; Friston, 2005; Mathys et al., 2014; Rao & Ballard, 1999). Within predictive coding, *beta oscillations* are assumed to convey top-down predictions, while *gamma oscillations* are thought to be involved in the bottom-up propagation of PEs (e.g. Arnal & Giraud, 2012; Bastos et al., 2012; Friston, Bastos, Pinotsis, & Litvak, 2015).

Combining this scheme with current proposals on beta synchronisation underlying the formation of large-scale distributed networks (NeuroCognitive Network, NCN; Bressler & Richter, 2015; Engel & Fries, 2010) and gamma synchronisation reflecting the matching of predicted and actual linguistic input during language processing (Lewis et al., 2015), Lewis and Bastiaansen (2015) hypothesised, that – within the language network – an increase in lower beta power signals the maintenance of its current configuration in case of effective construction of sentence-level meaning as well as resulting top-down directed transmission of predictions. By contrast, failed construction of sentence-level meaning, necessitating a change of the network configuration, would be reflected in a beta power decrease. An increase in middle gamma power, on the other hand, is assumed to indicate successful “matching” of predicted and actual linguistic input (no such change would be expected in case of a mismatch), while bottom-up directed PEs would be reflected in an increase in high gamma power. Respective findings, however, up to now are scarce and mainly stem from RSVP paradigms (see Meyer, 2018 for a recent review).

While there is some evidence for the hypothesised function of beta synchronisation for construction of sentence-level meaning (Lewis, Schoffelen, Schriefers, & Bastiaansen, 2016; Lewis et al., 2017), respective findings in terms of top-down predictions (rather than just “meaning”) are – to our knowledge – still pending. In fact, some recent studies have revealed somewhat contradictory findings rather suggesting that predictive processing during reading is reflected in *power suppression within the alpha and beta range*, critically, already prior to word onset, that is, within the pre-stimulus interval. To illustrate, Rommers, Dickson, Norton, Wlotko, and Federmeier (2017) could demonstrate that as sentence reading proceeded to the critical word, alpha power decreased when the word would be highly constrained by the sentence, resulting in a *decrease in pre-stimulus alpha power* (8–12 Hz) over occipital regions, indicating enhanced preparedness to process the anticipated input. Similar findings have been reported by Wang, Hagoort, and Jensen (2018a), showing a *decrease in pre-stimulus alpha* (8–12 Hz) and *beta power* (16–20 Hz) within a widespread network encompassing left inferior frontal and posterior temporal regions – including the Visual Word Form Area (VWFA; Cohen et al., 2002). Notably, the VWFA has been linked to visuo-orthographic processing, supposedly encoding whole-word recognition units (e.g. Kronbichler et al., 2004). Thus, the finding reported by Wang et al. (2018a) might not only – in line with Rommers et al. (2017) – indicate anticipatory engagement of language-related areas in general, but pre-activation of abstract visuo-orthographic word templates in particular (see also Willems, Frank, Nijhof, Hagoort, & van den Bosch, 2016).

With regard to the hypothesised functions of gamma oscillations, evidence is mixed. For example, while Wang et al. (2018a) indeed observed an *increase in gamma power* (60–90 Hz) over left temporal and frontal regions in response to semantically incongruent compared to congruent sentence-final words, this effect was accompanied by a *decrease in alpha power* (8–12 Hz) over left temporal and visual regions. Moreover, only alpha power decreases differed depending on sentential constraint, suggesting that an increase in gamma oscillations might not reflect PEs, but rather semantic unification and retrieval effort. Furthermore, within high constraining sentences, pre-stimulus alpha desynchronisation in temporal regions was negatively correlated with gamma power at stimulus onset in prefrontal regions suggesting a predictive network that utilises gamma for processing “correct predictions” rather than for purely error processing (see also Vidal et al., 2012; but see Penolazzi, Angrilli, & Job, 2009). This notion has been further substantiated by a follow-up study, indicating that gamma reflects the successful matching of predicted and actual input (Wang, Hagoort, & Jensen, 2018b). By contrast, Rommers et al. (2017) observed an *increase in frontal theta power* (4–7 Hz) for unexpected words in high constraining sentences, which, unlike Wang et al. (2018a), has been interpreted as increased requirement for cognitive control in case of failed predictions (see also Molinaro, Barraza, & Carreiras, 2013).

### Evidence from fixation-related oscillatory EEG

The first study investigating effects of predictability on fixation-related oscillatory dynamics – although not in the context of predictive coding – was done by Metzner et al. (2015), where the authors re-investigated previous RSVP findings which demonstrated not only robust N400 effects but also theta and gamma synchronisation in response to world knowledge violations (Hagoort et al., 2004). Interestingly, while Metzner and colleagues successfully replicated the N400 predictability effect both in ERPs and FRPs, an *increase in theta power* could only be found in the RSVP setting. During natural reading, however, the authors observed *delta-synchronisation* and *upper-alpha desynchronisation*.

A study explicitly evaluating Lewis and Bastiaansen’s framework was done by Vignali et al. (2016). Participants read syntactically well-formed and syntactically ill-formed sentences (sentences whose words were randomly shuffled), including either a semantically congruent or incongruent target word. In line with the theoretical assumptions of Lewis and Bastiaansen (2015), at the target-word Vignali and colleagues found a *desynchronisation in lower-beta* (13–18 Hz) only for semantically incongruent words, which may indicate how predicted words need re-updating – i.e. illustrating how a beta oscillation that carries an incorrect prediction requires disintegration. Interestingly to add to the gamma debate, the authors also reported an *increase in gamma power* (31–55 Hz) over the course of the sentences only for syntactically well-formed sentences as well as *higher theta power* (4–7 Hz) in well-formed compared to ill-formed sentences. In contrast to Metzner et al. (2015), this study therefore not only revealed qualitatively comparable results as the RSVP literature, but also modulations in gamma power on the sentence-level – a pattern that has also been observed with intracranial recordings and interpreted as construction of linguistic meaning (Fedorenko et al., 2016).

### Directed brain-connectivity to test for contextual predictions during natural reading

In light of only a few studies addressing the issue of contextual predictions and oscillatory network dynamics during (natural) reading, drawing firm conclusions with respect to its significance for linguistic processing would be premature. Still, the studies reviewed above demonstrate the potential of investigating oscillatory activity to identify how information is organised within the reading network and therefore to broaden our understanding about the “when and how” of contextual predictions in visual word recognition. It must be emphasised, however, that methods applied so far might only be suitable for testing *undirected network processing*, since they are indicative of functional (i.e. correlative) but not effective (i.e. causal) connectivity (see Friston, 2011 for a review). Furthermore, as described in the previous section, contextual effects during natural reading seem to be modulated by the availability of parafoveal information. Thus, it is plausible that oscillatory network dynamics are likewise sensitive to the predictability of upcoming information and its fit with parafoveal information.

Employing, for example, dynamic causal modelling (DCM; Chen, Kiebel, & Friston, 2008; Friston, Harrison, & Penny, 2003; Kiebel, Garrido, & Friston, 2007; Moran, Pinotsis, & Friston, 2013; Stephan et al., 2010) on co-registered EM and EEG data would allow us to test for effects of contextual predictions on *directed hierarchical information transmission* within the reading network (as, for example, suggested by Lewis & Bastiaansen, 2015) in an ecologically valid setting. We note that DCM has already successfully been applied to ERPs in the field of visual word recognition (e.g. Woodhead et al., 2014; Yvert, Perrone-Bertolotti, Baciu, & David, 2012) and EM behaviour in the context of Bayesian inference (e.g. Adams, Aponte, Marshall, & Friston, 2015; Adams, Bauer, Pinotsis, & Friston, 2016). Furthermore, DCM has provided fundamental insights into oscillatory dynamics underlying hierarchical processing as proposed by predictive coding theories of perception (e.g. Bastos et al., 2015). However some of these earlier predictions regarding the meaning of gamma oscillations for example, likely need revising in light of the oscillatory findings described here. Overall, the DCM approach not only offers the potential to investigate how top-down and bottom-up processing converges when encountering a specific word (e.g. pre-target versus target), but also how network dynamics underlying the transition from perception to comprehension evolve over time (e.g. from the beginning to the end of a sentence) while temporal and spatial aspects of EMs during natural reading are maintained. This is a crucial aspect with respect to the question of if and how we build up forward inferences during reading and, in consequence, whether contextual predictions during reading are mainly effective in prediction-encouraging tasks (Huettig & Mani, 2016), or are rather an inherent brain mechanism facilitating information processing during natural reading.

## Notes

1 We would like to point out that the discussion on predictive processing is nuanced and a comprehensive description would be beyond the scope of the article. For an in-depth discussion, we would like to refer the reader to Kuperberg and Jaeger (2016).

2 Word predictability is, traditionally, defined as the proportion [p] of readers (in an independent norming sample) predicting a particular word based on a previous sentence context in a so-called incremental cloze-task (Taylor, 1953). It must be noted that the utilisation of word predictability norms based on this procedure does not come without critique. In particular the circular mapping of behaviour to behaviour has been criticised to constitute a subjective rather than objective measure (Hofmann & Jacobs, 2014, p. 100). Moreover, the rather effortful collection of such predictability norms motivated attempts to approximate word predictability by means of corpus based transitional probabilities (e.g. McDonald & Shillcock, 2003). However, thorough investigations of the relation between effects of transitional probability and word predictability revealed that the variance in the eye movement parameters is better explained by predictability norms (Frisson, Rayner, & Pickering, 2005).

3 Lexical access is defined as the activation of a particular entry in the putative mental lexicon (Inhoff, 1984).

4 Word frequency is defined as the mean prevalence of a word in printed texts (Rayner, 1998, 2009). Please note that in this article we are primarily interested in the emergence of predictability effects in relation to the effect of word frequency. For a detailed analysis of the time-course of other linguistic variables (e.g. word length, orthographic similarity, semantic coherence) we would like to refer the reader to Hauk et al. (2006).

5 Saccade launch site is the takeoff point of a saccade. Saccade launch site distance is the distance between the takeoff point and the beginning of the aimed-at word (Heller & Müller, 1983).

## References

[CIT0001] Adams, R. A., Aponte, E., Marshall, L., & Friston, K. J. (2015). Active inference and oculomotor pursuit: The dynamic causal modelling of eye movements. *Journal of Neuroscience Methods*, 242, 1–14. 25583383 10.1016/j.jneumeth.2015.01.003 PMC4346275

[CIT0002] Adams, R. A., Bauer, M., Pinotsis, D., & Friston, K. J. (2016). Dynamic causal modelling of eye movements during pursuit: Confirming precision-encoding in V1 using MEG. *NeuroImage*, 132, 175–189. 26921713 10.1016/j.neuroimage.2016.02.055 PMC4862965

[CIT0003] Adams, R. A., Stephan, K. E., Brown, H. R., Frith, C. D., & Friston, K. J. (2013). The computational anatomy of psychosis. *Frontiers in Psychiatry*, 4, 47. 23750138 10.3389/fpsyt.2013.00047 PMC3667557

[CIT0004] Altarriba, J., Kroll, J. F., Sholl, A., & Rayner, K. (1996). The influence of lexical and conceptual constraints on reading mixed-language sentences: Evidence from eye fixations and naming times. *Memory & Cognition*, 24(4), 477–492. 8757496 10.3758/bf03200936

[CIT0005] Arnal, L. H., & Giraud, A. L. (2012). Cortical oscillations and sensory predictions. *Trends in Cognitive Sciences*, 16(7), 390–398. 22682813 10.1016/j.tics.2012.05.003

[CIT0006] Ashby, J., Rayner, K., & Clifton, C. (2005). Eye movements of highly skilled and average readers: Differential effects of frequency and predictability. *The Quarterly Journal of Experimental Psychology. A, Human Experimental Psychology*, 58(6), 1065–1086. 16194948 10.1080/02724980443000476

[CIT0007] Baccino, T. (2011). Eye movements and concurrent event-related potentials: Eye fixation-related potential investigations in reading. In S. P. Liversedge, I. D. Gilchrist, & S. Everling (Eds.), *Eye movements handbook* (pp. 857–870). Oxford: Oxford University Press.

[CIT0008] Baccino, T., & Manuta, Y. (2005). Eye-fixation-related potentials: Insight into parafoveal processing. *Journal of Psychophysiology*, 19, 204–215.

[CIT0009] Baggio, G. (2012). Selective alignment of brain responses by task demands during semantic processing. *Neuropsychologia*, 50(5), 655–665. 22245013 10.1016/j.neuropsychologia.2012.01.002

[CIT0010] Baggio, G., & Hagoort, P. (2011). The balance between memory and unification in semantics: A dynamic account of the N400. *Language and Cognitive Processes*, 26(9), 1338–1367.

[CIT0011] Balota, D. A., Pollatsek, A., & Rayner, K. (1985). The interaction of contextual constraints and parafoveal visual information in reading. *Cognitive Psychology*, 17(3), 364–390. 4053565 10.1016/0010-0285(85)90013-1

[CIT0012] Bassett, D. S., Meyer-Lindenberg, A., Achard, S., Duke, T., & Bullmore, E. (2006). Adaptive reconfiguration of fractal small-world human brain functional networks. *Proceedings of the National Academy of Sciences USA*, 103(51), 19518–19523. 10.1073/pnas.0606005103 PMC1838565 17159150

[CIT0013] Bastiaansen, M., & Hagoort, P. (2006). Oscillatory neuronal dynamics during language comprehension. *Progress in Brain Research*, 159, 179–196. 17071231 10.1016/S0079-6123(06)59012-0

[CIT0014] Bastos, A. M., Litvak, V., Moran, R., Bosman, C. A., Fries, P., & Friston, K. J. (2015). A DCM study of spectral asymmetries in feedforward and feedback connections between visual areas V1 and V4 in the monkey. *NeuroImage*, 108, 460–475. 25585017 10.1016/j.neuroimage.2014.12.081 PMC4334664

[CIT0015] Bastos, A. M., Usrey, W. M., Adams, R. A., Mangun, G. R., Fries, P., & Friston, K. J. (2012). Canonical microcircuits for predictive coding. *Neuron*, 76(4), 695–711. 23177956 10.1016/j.neuron.2012.10.038 PMC3777738

[CIT0016] Bonhage, C. E., Mueller, J. L., Friederici, A. D., & Fiebach, C. J. (2015). Combined eye tracking and fMRI reveals neural basis of linguistic predictions during sentence comprehension. *Cortex*, 68, 33–47. 26003489 10.1016/j.cortex.2015.04.011

[CIT0017] Bressler, S. L. (1995). Large-scale cortical networks and cognition. *Brain Research Reviews*, 20(3), 288–304. 7550362 10.1016/0165-0173(94)00016-i

[CIT0018] Bressler, S. L., & Richter, C. G. (2015). Interareal oscillatory synchronization in top-down neocortical processing. *Current Opinion in Neurobiology*, 31, 62–66. 25217807 10.1016/j.conb.2014.08.010

[CIT0019] Brothers, T., Swaab, T. Y., & Traxler, M. J. (2015). Effects of prediction and contextual support on lexical processing: Prediction takes precedence. *Cognition*, 136, 135–149. 25497522 10.1016/j.cognition.2014.10.017 PMC4308503

[CIT0020] Brouwer, A.-M., Reuderink, B., Vincent, J., van Gerven, M. A. J., & van Erp, J. B. F. (2013). Distinguishing between target and nontarget fixations in a visual search task using fixation-related potentials. *Journal of Vision*, 13(3), 17. 10.1167/13.3.17 23863335

[CIT0021] Buzsáki, G., & Draguhn, A. (2004). Neuronal oscillations in cortical networks. *Science*, 304(5679), 1926–1929. 15218136 10.1126/science.1099745

[CIT0022] Carreiras, M., Armstrong, B. C., Perea, M., & Frost, R. (2014). The what, when, where, and how of visual word recognition. *Trends in Cognitive Sciences*, 18(2), 90–98. 24373885 10.1016/j.tics.2013.11.005

[CIT0023] Chen, Y., Davis, M. H., Pulvermüller, F., & Hauk, O. (2015). Early visual word processing is flexible: evidence from spatiotemporal brain dynamics. *Journal of Cognitive Neuroscience*, 27(9), 1738–1751. 25848683 10.1162/jocn_a_00815

[CIT0024] Chen, C. C., Kiebel, S. J., & Friston, K. J. (2008). Dynamic causal modelling of induced responses. *Neuroimage*, 41(4), 1293–1312. 18485744 10.1016/j.neuroimage.2008.03.026

[CIT0025] Choi, W., Desai, R. H., & Henderson, J. M. (2014). The neural substrates of natural reading: A comparison of normal and nonword text using eyetracking and fMRI. *Frontiers in Human Neuroscience*, 8, 1024. 25566039 10.3389/fnhum.2014.01024 PMC4274877

[CIT0026] Clark, A. (2013). Whatever next? Predictive brains, situated agents, and the future of cognitive science. *Behavioral and Brain Sciences*, 36(3), 181–204. 10.1017/S0140525X12000477 23663408

[CIT0027] Cohen, M. X. (2017). Where does EEG come from and what does it mean? *Trends in Neurosciences*, 40(4), 208–218. 28314445 10.1016/j.tins.2017.02.004

[CIT0028] Cohen, L., Lehéricy, S., Chochon, F., Lemer, C., Rivaud, S., & Dehaene, S. (2002). Language-specific tuning of visual cortex? Functional properties of the visual word form area. *Brain*, 125(5), 1054–1069. 11960895 10.1093/brain/awf094

[CIT0029] Dale, A. M., & Buckner, R. L. (1997). Selective averaging of rapidly presented individual trials using fMRI. *Human Brain Mapping*, 5(5), 329–340. 20408237 10.1002/(SICI)1097-0193(1997)5:5<329::AID-HBM1>3.0.CO;2-5

[CIT0030] Dambacher, M., Dimigen, O., Braun, M., Wille, K., Jacobs, A. M., & Kliegl, R. (2012). Stimulus onset asynchrony and the timeline of word recognition: Event-related potentials during sentence reading. *Neuropsychologia*, 50(8), 1852–1870. 22564485 10.1016/j.neuropsychologia.2012.04.011

[CIT0031] Dambacher, M., Kliegl, R., Hofmann, M., & Jacobs, A. M. (2006). Frequency and predictability effects on event-related potentials during reading. *Brain Research*, 1084, 89–103. 16545344 10.1016/j.brainres.2006.02.010

[CIT0032] Dambacher, M., Rolfs, M., Göllner, K., Kliegl, R., & Jacobs, A. M. (2009). Event-related potentials reveal rapid verification of predicted visual input. *PLoS One*, 4, e5047. 19333386 10.1371/journal.pone.0005047 PMC2659434

[CIT0033] Degno, F., Loberg, O., Zang, C., Zhang, M., Donnelly, N., & Liversedge, S. P. (2018). Parafoveal previews and lexical frequency in natural reading: Evidence from eye movements and fixation-related potentials. *Journal of Experimental Psychology*, 148, 453–474. Epub ahead of print. 10.1037/xge0000494 30335444 PMC6388670

[CIT0034] DeLong, K. A., Troyer, M., & Kutas, M. (2014). Pre-processing in sentence comprehension: Sensitivity to likely upcoming meaning and structure. *Language and Linguistics Compass*, 8(12), 631–645. 27525035 10.1111/lnc3.12093 PMC4982702

[CIT0035] DeLong, K. A., Urbach, T. P., & Kutas, M. (2005). Probabilistic word pre-activation during language comprehension inferred from electrical brain activity. *Nature Neuroscience*, 8(8), 1117–1121. 16007080 10.1038/nn1504

[CIT0036] DeLong, K. A., Urbach, T. P., & Kutas, M. (2017). Is there a replication crisis? Perhaps. Is this an example? No: A commentary on Ito, Martin, and Nieuwland (2016). *Language, Cognition and Neuroscience*, 32(8), 966–973.

[CIT0037] Desai, R. H., Choi, W., Lai, V. T., & Henderson, J. M. (2016). Toward semantics in the wild: Activation to manipulable nouns in naturalistic reading. *The Journal of Neuroscience*, 36(14), 4050–4055. 27053211 10.1523/JNEUROSCI.1480-15.2016 PMC4821914

[CIT0038] Devillez, H., Guyader, N., & Guérin-Dugué, A. (2015). An eye fixation-related potentials analysis of the P300 potential for fixations onto a target object when exploring natural scenes. *Journal of Vision*, 15(13), 20. 10.1167/15.13.20 26401627

[CIT0039] Dimigen, O. (2018). Optimized ICA-based removal of ocular EEG artifacts from free viewing experiments. *bioRxiv*. 10.1101/446955

[CIT0040] Dimigen, O., Kliegl, R., & Sommer, W. (2012). Trans-saccadic parafoveal preview benefits in fluent reading: A study with fixation-related brain potentials. *Neuroimage*, 62(1), 381–393. 22521255 10.1016/j.neuroimage.2012.04.006

[CIT0041] Dimigen, O., Sommer, W., Dambacher, M., & Kliegl, R. (2008). Simultaneous recording of eye movements and ERPs indicates early access to word meaning in natural, left-to-right reading. *International Journal of Psychology*, 43(3–4), 47.

[CIT0042] Dimigen, O., Sommer, W., Hohlfeld, A., Jacobs, A. M., & Kliegl, R. (2011). Coregistration of eye movements and EEG in natural reading: Analyses and review. *Journal of Experimental Psychology: General*, 140(4), 552–572. 21744985 10.1037/a0023885

[CIT0043] Ehinger, B. V., & Dimigen, O. (2018). Unfold: An integrated toolbox for overlap correction, non-linear modeling, and regression-based EEG analysis. *bioRxiv*. 10.1101/360156

[CIT0044] Ehrlich, S. E., & Rayner, K. (1981). Contextual effects on word perception and eye movements during reading. *Journal of Verbal Learning and Verbal Behavior*, 20(6), 641–655.

[CIT0045] Engel, A. K., & Fries, P. (2010). Beta-band oscillations—Signalling the status quo? *Current Opinion in Neurobiology*, 20(2), 156–165. 20359884 10.1016/j.conb.2010.02.015

[CIT0046] Engel, A. K., Fries, P., & Singer, W. (2001). Dynamic predictions: Oscillations and synchrony in top-down processing. *Nature Reviews Neuroscience*, 2(10), 704–716. 11584308 10.1038/35094565

[CIT0047] Federmeier, K. D., & Kutas, M. (1999). A rose by any other name: Long-term memory structure and sentence processing. *Journal of Memory and Language*, 41(4), 469–495.

[CIT0048] Fedorenko, E., Scott, T. L., Brunner, P., Coon, W. G., Pritchett, B., Schalk, G., & Kanwisher, N. (2016). Neural correlate of the construction of sentence meaning. *Proceedings of the National Academy of Sciences*, 113(41), E6256–E6262. 10.1073/pnas.1612132113 PMC5068329 27671642

[CIT0049] Finke, A., Essig, K., Marchioro, G., & Ritter, H. (2016). Toward FRP-based brain-machine interfaces—Single-trial classification of fixation-related potentials. *PLoS One*, 11(1), e0146848. 26812487 10.1371/journal.pone.0146848 PMC4727887

[CIT0050] Fischer, T., Graupner, S.-T., Velichkovsky, B. M., & Pannasch, S. P. (2013). Attentional dynamics during free picture viewing: Evidence from oculomotor behavior and electrocortical activity. *Frontiers in Systems Neuroscience*, 7, 17. 23759704 10.3389/fnsys.2013.00017 PMC3671178

[CIT0051] Fitzsimmons, G., & Drieghe, D. (2013). How fast can predictability influence word skipping during reading? *Journal of Experimental Psychology: Learning, Memory, and Cognition*, 39(4), 1054–1063. 10.1037/a0030909 23244054

[CIT0052] Frisson, S., Rayner, K., & Pickering, M. J. (2005). Effects of contextual predictability and transitional probability on eye movements during reading. *Journal of Experimental Psychology: Learning, Memory, and Cognition*, 31(5), 862–877. 10.1037/0278-7393.31.5.862 16248738

[CIT0053] Friston, K. J. (2005). A theory of cortical responses. *Philosophical Transactions of the Royal Society B: Biological Sciences*, 360(1456), 815–836. 10.1098/rstb.2005.1622 PMC1569488 15937014

[CIT0054] Friston, K. J. (2009). The free-energy principle: A rough guide to the brain? *Trends in Cognitive Sciences*, 13(7), 293–301. 19559644 10.1016/j.tics.2009.04.005

[CIT0055] Friston, K. J. (2010). The free-energy principle: A unified brain theory? *Nature Reviews Neuroscience*, 11, 127–138. 20068583 10.1038/nrn2787

[CIT0056] Friston, K. J. (2011). Functional and effective connectivity: A review. *Brain Connectivity*, 1(1), 13–36. 22432952 10.1089/brain.2011.0008

[CIT0057] Friston, K. J., Bastos, A. M., Pinotsis, D., & Litvak, V. (2015). LFP and oscillations—What do they tell us? *Current Opinion in Neurobiology*, 31, 1–6. 25079053 10.1016/j.conb.2014.05.004 PMC4376394

[CIT0058] Friston, K. J., Harrison, L., & Penny, W. (2003). Dynamic causal modelling. *Neuroimage*, 19(4), 1273–1302. 12948688 10.1016/s1053-8119(03)00202-7

[CIT0059] Hagoort, P., Hald, L., Bastiaansen, M., & Petersson, K. M. (2004). Integration of word meaning and world knowledge in language comprehension. *Science*, 304(5669), 438–441. 15031438 10.1126/science.1095455

[CIT0060] Haller, M., Donoghue, T., Peterson, E., Varma, P., Sebastian, P., Gao, R., & Voytek, B. (2018). Parameterizing neural power spectra. *bioRxiv*. 10.1101/299859

[CIT0061] Hand, C. J., Miellet, S., O’Donnell, P. J., & Sereno, S. C. (2010). The frequency-predictability interaction in reading: It depends where you’re coming from. *Journal of Experimental Psychology: Human Perception and Performance*, 36(5), 1294–1313. 20854004 10.1037/a0020363

[CIT0062] Harley, T. (2014). *The psychology of language* (4th ed). London: Psychology Press.

[CIT0063] Hauk, O., Davis, M. H., Ford, M., Pulvermuller, F., & Marslen-Wilson, W. D. (2006). The time course of visual word recognition as revealed by linear regression analysis of ERP data. *Neuroimage*, 30(4), 1383–1400. 16460964 10.1016/j.neuroimage.2005.11.048

[CIT0064] Hauk, O., & Pulvermüller, F. (2004). Effects of word length and frequency on the human event-related potential. *Clinical Neurophysiology*, 115, 1090–1103. 15066535 10.1016/j.clinph.2003.12.020

[CIT0065] Hawelka, S., Schuster, S., Gagl, B., & Hutzler, F. (2015). On forward inferences of fast and slow readers. An eye movement study. *Scientific Reports*, 5, 8432. 25678030 10.1038/srep08432 PMC4327408

[CIT0066] Heller, D., & Müller, H. (1983). On the relationship of saccade size and fixation duration in reading. In R. Groner, C. Menz, D. F. Fisher, & R. A. Monty (Eds.), *Eye movements and psychological functions: International views* (pp. 287–302). Hillsdale, NJ: Erlbaum.

[CIT0067] Henderson, J. M., & Choi, W. (2015). Neural correlates of fixation duration during Real-world scene viewing: Evidence from fixation-related (FIRE) fMRI. *Journal of Cognitive Neuroscience*, 27(6), 1137–1145. 25436668 10.1162/jocn_a_00769

[CIT0068] Henderson, J. M., Choi, W., Lowder, M. W., & Ferreira, F. (2016). Language structure in the brain: A fixation-related fMRI study of syntactic surprisal in reading. *Neuroimage*, 132, 293–300. 26908322 10.1016/j.neuroimage.2016.02.050

[CIT0069] Henderson, J. M., Choi, W., Luke, S. G., & Desai, R. H. (2015). Neural correlates of fixation duration in natural reading: Evidence from fixation-related fMRI. *Neuroimage*, 119, 390–397. 26151101 10.1016/j.neuroimage.2015.06.072

[CIT0070] Henderson, J. M., Choi, W., Luke, S. G., & Schmidt, J. (2018). Neural correlates of individual differences in fixation duration during natural reading. *Quarterly Journal of Experimental Psychology (Hove)*, 71, 314–323. 10.1080/17470218.2017.1329322 28508716

[CIT0071] Henderson, J. M., & Ferreira, F. (1993). Eye movement control during reading: Fixation measures reflect foveal but not parafoveal processing difficulty. *Canadian Journal of Experimental Psychology/Revue Canadienne de Psychologie Expérimentale*, 47(2), 201–221. 8364530 10.1037/h0078814

[CIT0072] Henderson, J. M., Luke, S. G., Schmidt, J., & Richards, J. E. (2013). Co-registration of eye movements and event-related potentials in connected-text paragraph reading. *Frontiers in Systems Neuroscience*, 7, 28. 23847477 10.3389/fnsys.2013.00028 PMC3706749

[CIT0073] Hipp, J. F., Engel, A. K., & Siegel, M. (2011). Oscillatory synchronization in large-scale cortical networks predicts perception. *Neuron*, 69(2), 387–396. 21262474 10.1016/j.neuron.2010.12.027

[CIT0074] Hofmann, M. J., & Jacobs, A. M. (2014). Interactive activation and competition models and semantic context: From behavioral to brain data. *Neuroscience & Biobehavioral Reviews*, 46(1), 85–104. 24992217 10.1016/j.neubiorev.2014.06.011

[CIT0075] Huettig, F. (2015). Four central questions about prediction in language processing. *Brain Research*, 1626, 118–135. 25708148 10.1016/j.brainres.2015.02.014

[CIT0076] Huettig, F., & Mani, N. (2016). Is prediction necessary to understand language? Probably not. *Language, Cognition and Neuroscience*, 31(1), 19–31.

[CIT0077] Hutzler, F., Braun, M., Võ, M. L., Engl, V., Hofmann, M., Dambacher, M., & Jacobs, A. M. (2007). Welcome to the real world: Validating fixation-related brain potentials for ecologically valid settings. *Brain Research*, 1172, 124–129. 17803976 10.1016/j.brainres.2007.07.025

[CIT0078] Hutzler, F., Fuchs, I., Gagl, B., Schuster, S., Richlan, F., Braun, M., & Hawelka, S. (2013). Parafoveal X-masks interfere with foveal word recognition: Evidence from fixation-related brain potentials. *Frontiers in Systems Neuroscience*, 7, 33. 23888130 10.3389/fnsys.2013.00033 PMC3719217

[CIT0079] Hyönä, J., & Olson, R. K. (1995). Eye fixation patterns among dyslexic and normal readers: Effects of word length and word frequency. *Journal of Experimental Psychology: Learning, Memory, and Cognition*, 21(6), 1430–1440. 10.1037//0278-7393.21.6.1430 7490575

[CIT0080] Inhoff, A. W. (1984). Two stages of word processing during eye fixations in the reading of prose. *Journal of Verbal Learning and Verbal Behavior*, 23(5), 612–624.

[CIT0081] Inhoff, A. W., & Rayner, K. (1986). Parafoveal word processing during eye fixations in reading: Effects of word frequency. *Perception & Psychophysics*, 40(6), 431–439. 3808910 10.3758/bf03208203

[CIT0082] Ito, A., Martin, A. E., & Nieuwland, M. S. (2017a). How robust are prediction effects in language comprehension? Failure to replicate article-elicited N400 effects. *Language, Cognition and Neuroscience*, 32(8), 954–965.

[CIT0083] Ito, A., Martin, A. E., & Nieuwland, M. S. (2017b). Why the A/AN prediction effect may be hard to replicate: A rebuttal to Delong, Urbach, and Kutas (2017). *Language, Cognition and Neuroscience*, 32(8), 974–983.

[CIT0084] Jobard, G., Crivello, F., & Tzourio-Mazoyer, N. (2003). Evaluation of the dual route theory of reading: A metanalysis of 35 neuroimaging studies. *Neuroimage*, 20(2), 693–712. 14568445 10.1016/S1053-8119(03)00343-4

[CIT0085] Kamienkowski, J. E., Ison, M. J., Quiroga, R. Q., & Sigman, M. (2012). Fixation-related potentials in visual search: A combined EEG and eye tracking study. *Journal of Vision*, 12(7), 4. 10.1167/12.7.4 22776848

[CIT0086] Kaunitz, L. N., Kamienkowski, J. E., Varatharajah, A., Sigman, M., Quiroga, R. Q., & Ison, M. J. (2014). Looking for a face in the crowd: Fixation-related potentials in an eye-movement visual search task. *Neuroimage*, 89, 297–305. 24342226 10.1016/j.neuroimage.2013.12.006

[CIT0087] Kiebel, S. J., Garrido, M. I., & Friston, K. J. (2007). Dynamic causal modelling of evoked responses: The role of intrinsic connections. *Neuroimage*, 36(2), 332–345. 17462916 10.1016/j.neuroimage.2007.02.046

[CIT0088] King, J. W., & Kutas, M. (1998). Neural plasticity in the dynamics of human visual word recognition. *Neuroscience Letters*, 244, 61–64. 9572585 10.1016/s0304-3940(98)00140-2

[CIT0089] Kliegl, R., Dambacher, M., Dimigen, O., Jacobs, A. M., & Sommer, W. (2012). Eye movements and brain electric potentials during reading. *Psychological Research*, 76(2), 145–158. 21915693 10.1007/s00426-011-0376-x

[CIT0090] Kliegl, R., Dambacher, M., Dimigen, O., & Sommer, W. (2014). Oculomotor control, brain potentials, and timelines of word recognition during natural reading. In M. Horsley, M. Eliot, B. Knight, & R. Reilly (Eds.), *Current trends in eye tracking research* (pp. 141–155). Berlin, Germany: Springer.

[CIT0091] Kliegl, R., Grabner, E., Rolfs, E., & Engbert, R. (2004). Length, frequency, and predictability effects of words on eye movements in reading. *European Journal of Cognitive Psychology*, 16(1–2), 262–284.

[CIT0092] Kliegl, R., Hohenstein, S., Yan, M., & McDonald, S. A. (2013). How preview space/time translates into preview cost/benefit for fixation durations during reading. *Quarterly Journal of Experimental Psychology (Hove)*, 66(3), 581–600. 10.1080/17470218.2012.658073 22515948

[CIT0093] Kliegl, R., Nuthmann, A., & Engbert, R. (2006). Tracking the mind during reading: The influence of past, present, and future words on fixation durations. *Journal of Experimental Psychology: General*, 135(1), 12–35. 16478314 10.1037/0096-3445.135.1.12

[CIT0094] Klimesch, W., Sauseng, P., & Hanslmayr, S. (2007). EEG alpha oscillations: The inhibition–timing hypothesis. *Brain Research Reviews*, 53(1), 63–88. 16887192 10.1016/j.brainresrev.2006.06.003

[CIT0095] Körner, C., Braunstein, V., Stangl, M., Schlögl, A., Neuper, C., & Ischebeck, A. (2014). Sequential effects in continued visual search: Using fixation-related potentials to compare distractor processing before and after target detection. *Psychophysiology*, 51(4), 385–395. 24512467 10.1111/psyp.12062 PMC4283708

[CIT0096] Kornrumpf, B., Dimigen, O., & Sommer, W. (2017). Lateralization of posterior alpha EEG reflects the distribution of spatial attention during saccadic reading. *Psychophysiology*, 54(6), 809–823. 28240816 10.1111/psyp.12849

[CIT0097] Kornrumpf, B., Niefind, F., Sommer, W., & Dimigen, O. (2016). Neural correlates of word recognition: A Systematic comparison of natural reading and rapid serial visual presentation. *Journal of Cognitive Neuroscience*, 28(9), 1374–1391. 27167402 10.1162/jocn_a_00977

[CIT0098] Kretzschmar, F., Bornkessel-Schlesewsky, I., & Schlesewsky, M. (2009). Parafoveal versus foveal N400s dissociate spreading activation from contextual fit. *Neuroreport*, 20(18), 1613–1618. 19884865 10.1097/WNR.0b013e328332c4f4

[CIT0099] Kretzschmar, F., Schlesewsky, M., & Staub, A. (2015). Dissociating word frequency and predictability effects in reading: Evidence from coregistration of eye movements and EEG. *Journal of Experimental Psychology: Learning, Memory, and Cognition*, 41(6), 1648–1662. 10.1037/xlm0000128 26010829

[CIT0100] Kronbichler, M., Hutzler, F., Wimmer, H., Mair, A., Staffen, W., & Ladurner, G. (2004). The visual word form area and the frequency with which words are encountered: Evidence from a parametric fMRI study. *Neuroimage*, 21(3), 946–953. 15006661 10.1016/j.neuroimage.2003.10.021

[CIT0101] Kuperberg, G. R. (2016). Separate streams or probabilistic inference? What the N400 can tell us about the comprehension of events. *Language, Cognition and Neuroscience*, 31(5), 602–616. 10.1080/23273798.2015.1130233 PMC4996121 27570786

[CIT0102] Kuperberg, G. R., & Jaeger, T. F. (2016). What do we mean by prediction in language comprehension? *Language, Cognition and Neuroscience*, 31(1), 32–59. 10.1080/23273798.2015.1102299 PMC4850025 27135040

[CIT0103] Kuperberg, G. R., Kreher, D. A., & Ditman, T. (2010). What can event-related potentials tell us about language, and perhaps even thought, in schizophrenia? *International Journal of Psychophysiology*, 75(2), 66–76. 19765622 10.1016/j.ijpsycho.2009.09.005 PMC3136365

[CIT0104] Kuperberg, G. R., Sitnikova, T., Caplan, D., & Holcomb, P. J. (2003). Electrophysiological distinctions in processing conceptual relationships within simple sentences. *Brain Research. Cognitive Brain Research*, 17(1), 117–129. 12763198 10.1016/s0926-6410(03)00086-7

[CIT0105] Kutas, M., DeLong, K. A., & Smith, N. J. (2011). A look around at what lies ahead: Prediction and predictability in language processing. In M. Bar (Ed.), *Predictions in the brain: Using our past to generate a future* (pp. 190–207). New York, NY: Oxford University Press.

[CIT0106] Kutas, M., & Federmeier, K. D. (2000). Electrophysiology reveals semantic memory use in language comprehension. *Trends in Cognitive Sciences*, 4(12), 463–470. 11115760 10.1016/s1364-6613(00)01560-6

[CIT0107] Kutas, M., & Federmeier, K. D. (2011). Thirty years and counting: Finding meaning in the N400 component of the event-related brain potential (ERP). *Annual Review of Psychology*, 62, 621–647. 10.1146/annurev.psych.093008.131123 PMC4052444 20809790

[CIT0108] Kutas, M., & Hillyard, S. A. (1980). Reading senseless sentences: Brain potentials reflect semantic incongruity. *Science*, 207(4427), 203–205. 7350657 10.1126/science.7350657

[CIT0109] Kutas, M., & Hillyard, S. A. (1984). Brain potentials during reading reflect word expectancy and semantic association. *Nature*, 307(5947), 161–163. 6690995 10.1038/307161a0

[CIT0110] Laszlo, S., & Federmeier, K. D. (2014). Never seem to find the time: Evaluating the physiological time course of visual word recognition with regression analysis of single-item event-related potentials. *Language, Cognition and Neuroscience*, 29, 642–661. 10.1080/01690965.2013.866259 PMC4060970 24954966

[CIT0111] Lau, E. F., Phillips, C., & Poeppel, D. (2008). A cortical network for semantics: (de)constructing the N400. *Nature Reviews Neuroscience*, 9(12), 920–933. 19020511 10.1038/nrn2532

[CIT0112] Lavigne, F., Vitu, F., & d’Ydewalle, G. (2000). The influence of semantic context on initial eye landing sites in words. *Acta Psychologica*, 104(2), 191–214. 10900705 10.1016/s0001-6918(00)00020-2

[CIT0113] Lee, C.-Y., Liu, Y.-N., & Tsai, J.-L. (2012). The time-course of contextual effects on visual word recognition. *Frontiers in Psychology*, 3, 1–13. 22934087 10.3389/fpsyg.2012.00285 PMC3422729

[CIT0114] Lewis, A. G., & Bastiaansen, M. (2015). A predictive coding framework for rapid neural dynamics during sentence-level language comprehension. *Cortex*, 68, 155–168. 25840879 10.1016/j.cortex.2015.02.014

[CIT0115] Lewis, A. G., Schoffelen, J.-M., Hoffmann, C., Bastiaansen, M., & Schriefers, H. (2017). Discourse-level semantic coherence influences beta oscillatory dynamics and the N400 during sentence comprehension. *Language, Cognition and Neuroscience*, 32(5), 601–617.

[CIT0116] Lewis, A. G., Schoffelen, J.-M., Schriefers, H., & Bastiaansen, M. (2016). A predictive coding perspective on beta oscillations during sentence-level language comprehension. *Frontiers in Human Neuroscience*, 10, 85. 26973500 10.3389/fnhum.2016.00085 PMC4776303

[CIT0117] Lewis, A. G., Wang, L., & Bastiaansen, M. (2015). Fast oscillatory dynamics during language comprehension: Unification versus maintenance and prediction? *Brain and Language*, 148, 51–63. 25666170 10.1016/j.bandl.2015.01.003

[CIT0118] Marsman, J. B., Renken, R., Velichkovsky, B. M., Hooymans, J. M., & Cornelissen, F. W. (2012). Fixation based event-related fMRI analysis: Using eye fixations as events in functional magnetic resonance imaging to reveal cortical processing during the free exploration of visual images. *Human Brain Mapping*, 33(2), 307–318. 21472819 10.1002/hbm.21211 PMC6870201

[CIT0119] Martin, C. D., Thierry, G., Kuipers, J.-R., Boutonnet, B., Foucart, A., & Costa, A. (2013). Bilinguals reading in their second language do not predict upcoming words as native readers do. *Journal of Memory and Language*, 69(4), 574–588.

[CIT0120] Marx, C., Hawelka, S., Schuster, S., & Hutzler, F. (2015). An incremental boundary study on parafoveal preprocessing in children reading aloud: Parafoveal masks overestimate the preview benefit. *Journal of Cognitive Psychology (Hove)*, 27(5), 549–561. 10.1080/20445911.2015.1008494 PMC4487581 26246890

[CIT0121] Mathys, C. D., Lomakina, E. I., Daunizeau, J., Iglesias, S., Brodersen, K. H., Friston, K. J., & Stephan, K. E. (2014). Uncertainty in perception and the hierarchical Gaussian filter. *Frontiers in Human Neuroscience*, 8, 825. 25477800 10.3389/fnhum.2014.00825 PMC4237059

[CIT0122] McDonald, S. A., & Shillcock, R. C. (2003). Eye movements reveal the on-line computation of lexical probabilities during reading. *Psychological Science*, 14(6), 648–652. 14629701 10.1046/j.0956-7976.2003.psci_1480.x

[CIT0123] Metzner, P., von der Malsburg, T., Vasishth, S., & Rösler, F. (2015). Brain responses to world knowledge violations: A comparison of stimulus- and fixation-triggered event-related potentials and neural oscillations. *Journal of Cognitive Neuroscience*, 27(5), 1017–1028. 25269112 10.1162/jocn_a_00731

[CIT0124] Metzner, P., von der Malsburg, T., Vasishth, S., & Rösler, F. (2017). The importance of reading naturally: Evidence from combined recordings of eye movements and electric brain potentials. *Cognitive Science*, 41(6), 1232–1263. 27307404 10.1111/cogs.12384

[CIT0125] Meyer, L. (2018). The neural oscillations of speech processing and language comprehension: State of the art and emerging mechanisms. *European Journal of Neuroscience*, 48(7), 2609–2621. 10.1111/ejn.13748 29055058

[CIT0126] Miellet, S., Sparrow, L., & Sereno, S. C. (2007). Word frequency and predictability effects in reading French: An evaluation of the E-Z reader model. *Psychonomic Bulletin & Review*, 14(4), 762–769. 17972746 10.3758/bf03196834

[CIT0127] Molinaro, N., Barraza, P., & Carreiras, M. (2013). Long-range neural synchronization supports fast and efficient reading: EEG correlates of processing expected words in sentences. *NeuroImage*, 72, 120–132. 23357072 10.1016/j.neuroimage.2013.01.031 PMC3817365

[CIT0128] Moran, R., Pinotsis, D. A., & Friston, K. (2013). Neural masses and fields in dynamic causal modeling. *Frontiers in Computational Neuroscience*, 7, 57. 23755005 10.3389/fncom.2013.00057 PMC3664834

[CIT0129] Niefind, F., & Dimigen, O. (2016). Dissociating parafoveal preview benefit and parafovea-on-fovea effects during reading: A combined eye tracking and EEG study. *Psychophysiology*, 53(12), 1784–1798. 27680711 10.1111/psyp.12765

[CIT0130] Nieuwland, M. S., Barr, D. J., Bartolozzi, F., Busch-Moreno, S., Darley, E., Donaldson, D. I., … Von Grebmer Zu Wolfsthurn, S. (2018a). Dissociable effects of prediction and integration during language comprehension: Evidence from a large-scale study using brain potentials. *bioRxiv*. 10.1101/267815

[CIT0131] Nieuwland, M. S., Politzer-Ahles, S., Heyselaar, E., Segaert, K., Darley, E., Kazanina, N., … Huettig, F. (2018b). Limits on prediction in language comprehension: A multi-lab failure to replicate evidence for probabilistic pre-activation of phonology. *eLife*, 7, e33468.29631695

[CIT0132] Nikolaev, A. R., Jurica, P., Nakatani, C., Plomp, G., & van Leeuwen, C. (2013). Visual encoding and fixation target selection in free viewing: Presaccadic brain potentials. *Frontiers in System Neuroscience*, 7, 26. 10.3389/fnsys.2013.00026 PMC3694272 23818877

[CIT0133] Nikolaev, A. R., Nakatani, C., Plomp, G., Jurica, P., & van Leeuwen, C. (2011). Eye fixation-related potentials in free viewing identify encoding failures in change detection. *Neuroimage*, 56(3), 1598–1607. 21406236 10.1016/j.neuroimage.2011.03.021

[CIT0134] Ossandón, J. P., Helo, A. V., Montefusco-Siegmund, R., & Maldonado, P. E. (2010). Superposition model Predicts EEG occipital activity during free viewing of natural Scenes. *The Journal of Neuroscience*, 30(13), 4787–4795. 20357129 10.1523/JNEUROSCI.5769-09.2010 PMC6632312

[CIT0135] Penolazzi, B., Angrilli, A., & Job, R. (2009). Gamma EEG activity induced by semantic violation during sentence reading. *Neuroscience Letters*, 465, 74–78. 19723559 10.1016/j.neulet.2009.08.065

[CIT0136] Penolazzi, B., Hauk, O., & Pulvermüller, F. (2007). Early semantic context integration and lexical access as revealed by event-related brain potentials. *Biological Psychology*, 74(3), 374–388. 17150298 10.1016/j.biopsycho.2006.09.008

[CIT0137] Plöchl, M., Ossandón, J. P., & König, P. (2012). Combining EEG and eye tracking: Identification, characterization, and correction of eye movement artifacts in electroencephalographic data. *Frontiers in Human Neuroscience*, 6, 278. 23087632 10.3389/fnhum.2012.00278 PMC3466435

[CIT0138] Price, C. J. (2012). A review and synthesis of the first 20 years of PET and fMRI studies of heard speech, spoken language and reading. *Neuroimage*, 62(2), 816–847. 22584224 10.1016/j.neuroimage.2012.04.062 PMC3398395

[CIT0139] Rao, R. P., & Ballard, D. H. (1999). Predictive coding in the visual cortex: A functional interpretation of some extra-classical receptive-field effects. *Nature Neuroscience*, 2(1), 79–87. 10195184 10.1038/4580

[CIT0140] Rauss, K., & Pourtois, G. (2013). What is bottom-up and what is top-down in predictive coding? *Frontiers in Psychology*, 4, 276. 23730295 10.3389/fpsyg.2013.00276 PMC3656342

[CIT0141] Rayner, K. (1975). The perceptual span and peripheral cues in reading. *Cognitive Psychology*, 7(1), 65–81.

[CIT0142] Rayner, K. (1998). Eye movements in reading and information processing: 20 years of research. *Psychological Bulletin*, 124(3), 372–422. 9849112 10.1037/0033-2909.124.3.372

[CIT0143] Rayner, K. (2009). The 35th Sir Frederick Bartlett Lecture: Eye movements and attention in reading, scene perception, and visual search. *Quarterly Journal of Experimental Psychology (Hove)*, 62(8), 1457–1506. 10.1080/17470210902816461 19449261

[CIT0144] Rayner, K., Ashby, J., Pollatsek, A., & Reichle, E. D. (2004). The effects of frequency and predictability on eye fixations in reading: Implications for the E-Z reader model. *Journal of Experimental Psychology: Human Perception and Performance*, 30(4), 720–732. 15301620 10.1037/0096-1523.30.4.720

[CIT0145] Rayner, K., Binder, K. S., Ashby, J., & Pollatsek, A. (2001). Eye movement control in reading: Word predictability has little influence on initial landing positions in words. *Vision Research*, 41(7), 943–954. 11248279 10.1016/s0042-6989(00)00310-2

[CIT0146] Rayner, K., & Duffy, S. A. (1986). Lexical complexity and fixation times in reading: Effects of word frequency, verb complexity, and lexical ambiguity. *Memory & Cognition*, 14(3), 191–201. 3736392 10.3758/bf03197692

[CIT0147] Rayner, K., Sereno, S. C., Morris, R. K., Schmauder, A. R., & Clifton, C. (1989). Eye movements and on-line language comprehension processes. *Language and Cognition Processes*, 4(3–4), 21–49.

[CIT0148] Rayner, K., Sereno, S. C., & Raney, G. E. (1996). Eye movement control in reading: A comparison of two types of models. *Journal of Experimental Psychology Human Perception & Performance*, 22(5), 1188–1200. 8865619 10.1037//0096-1523.22.5.1188

[CIT0149] Rayner, K., & Well, A. D. (1996). Effects of contextual constraint on eye movements in reading: A further examination. *Psychonomic Bulletin & Review*, 3(4), 504–509. 24213985 10.3758/BF03214555

[CIT0150] Reichle, E. D., Tokowicz, N., Liu, Y., & Perfetti, C. A. (2011). Testing an assumption of the E-Z reader model of eye-movement control during reading: Using event-related potentials to examine the familiarity check. *Psychophysiology*, 48(7), 993–1003. 21261631 10.1111/j.1469-8986.2011.01169.x

[CIT0151] Reilly, R. G. (2014). Triangulating the reading brain: Eye movements, computational models, and EEG. In M. Horsley, M. Eliot, B. A. Knight, & R. G. Reilly (Eds.), *Current trends in eye tracking research* (pp. 131–139). New York, NY: Springer.

[CIT0152] Reingold, E. M., Reichle, E. D., Glaholt, M. G., & Sheridan, H. (2012). Direct lexical control of eye movements in reading: Evidence from a survival analysis of fixation durations. *Cognitive Psychology*, 65(2), 177–206. 22542804 10.1016/j.cogpsych.2012.03.001 PMC3565237

[CIT0153] Richlan, F., Gagl, B., Hawelka, S., Braun, M., Schurz, M., Kronbichler, M., & Hutzler, F. (2014). Fixation-related fMRI analysis in the domain of reading research: Using self-paced eye movements as markers for hemodynamic brain responses during visual letter string processing. *Cerebral Cortex*, 24(10), 2647–2656. 23645718 10.1093/cercor/bht117 PMC4153805

[CIT0154] Richlan, F., Gagl, B., Schuster, S., Hawelka, S., Humberger, J., & Hutzler, F. (2013). A new high-speed visual stimulation method for gaze-contingent eye movement and brain activity studies. *Frontiers in Systems Neuroscience*, 7, 24. 23847475 10.3389/fnsys.2013.00024 PMC3696721

[CIT0155] Rommers, J., Dickson, D. S., Norton, J. J. S., Wlotko, E. W., & Federmeier, K. D. (2017). Alpha and theta band dynamics related to sentential constraint and word expectancy. *Language, Cognition and Neuroscience*, 32(5), 576–589. 10.1080/23273798.2016.1183799 PMC5533299 28761896

[CIT0156] Rugg, M. D. (1990). Event-related brain potentials dissociate repetition effects of high-and low-frequency words. *Memory & Cognition*, 18, 367–379. 2381316 10.3758/bf03197126

[CIT0157] Schilling, H. H., Rayner, K., & Chumbley, J. I. (1998). Comparing naming, lexical decision, and eye fixation times: Word frequency effects and individual differences. *Memory & Cognition*, 26(6), 1270–1281. 9847550 10.3758/bf03201199

[CIT0158] Schotter, E. R., Tran, R., & Rayner, K. (2014). Don’t believe what you read (only once): comprehension is supported by regressions during reading. *Psychological Science*, 25(6), 1218–1226. 24747167 10.1177/0956797614531148

[CIT0159] Schuster, S., Hawelka, S., Himmelstoss, N. A., Richlan, F., & Hutzler, F. (in press). The neural correlates of word position and lexical predictability during sentence reading: Evidence from fixation-related fMRI. *Language, Cognition and Neuroscience*. doi:10.1080/23273798.2019.1575970.

[CIT0160] Schuster, S., Hawelka, S., Hutzler, F., Kronbichler, M., & Richlan, F. (2016). Words in context: The effects of length, frequency, and predictability on brain responses during natural reading. *Cerebral Cortex*, 26(10), 3889–3904. 10.1093/cercor/bhw184 PMC5028003 27365297

[CIT0161] Schuster, S., Hawelka, S., Richlan, F., Ludersdorfer, P., & Hutzler, F. (2015). Eyes on words: A fixation-related fMRI study of the left occipito-temporal cortex during self-paced silent reading of words and pseudowords. *Scientific Reports*, 5, 12686. 26235228 10.1038/srep12686 PMC4522675

[CIT0162] Sereno, S. C., Brewer, C. C., & O’Donnell, P. J. (2003). Context effects in word recognition: Evidence for early interactive processing. *Psychological Science*, 14(4), 328–333. 12807405 10.1111/1467-9280.14471

[CIT0163] Sereno, S. C., Hand, C. J., Shahid, A., Yao, B., & O’Donnell, P. J. (2018). Testing the limits of contextual constraint: Interactions with word frequency and parafoveal preview during fluent reading. *Quarterly Journal of Experimental Psychology (Hove)*, 71(1), 302–313. 10.1080/17470218.2017.1327981 PMC6159772 28481189

[CIT0164] Sereno, S. C., & Rayner, K. (2003). Measuring word recognition in reading: Eye movements and event-related potentials. *Trends in Cognitive Sciences*, 7(11), 489–493. 14585445 10.1016/j.tics.2003.09.010

[CIT0165] Sereno, S. C., Rayner, K., & Posner, M. I. (1998). Establishing a time-line of word recognition: Evidence from eye movements and event-related potentials. *Neuroreport*, 9(10), 2195–2200. 9694199 10.1097/00001756-199807130-00009

[CIT0166] Sheridan, H., & Reingold, E. M. (2012). The time course of contextual influences during lexical ambiguity resolution: Evidence from distributional analyses of fixation durations. *Memory & Cognition*, 40(7), 1122–1131. 22576974 10.3758/s13421-012-0216-2

[CIT0167] Simola, J., Holmqvist, K., & Lindgren, M. (2009). Right visual field advantage in parafoveal processing: Evidence from eye-fixation-related potentials. *Brain and Language*, 111(2), 101–113. 19782390 10.1016/j.bandl.2009.08.004

[CIT0168] Slattery, T. J., Pollatsek, A., & Rayner, K. (2007). The effect of the frequencies of three consecutive content words on eye movements during reading. *Memory & Cognition*, 35(6), 1283–1292. 18035627 10.3758/bf03193601

[CIT0169] Slattery, T. J., Staub, A., & Rayner, K. (2012). Saccade launch site as a predictor of fixation durations in reading: Comments on Hand, Miellet, O’Donnell, and Sereno (2010). *Journal of Experimental Psychology: Human Perception and Performance*, 38(1), 251–261. 22082213 10.1037/a0025980

[CIT0170] Staub, A. (2015). The effect of lexical predictability on eye movements in reading: Critical review and theoretical interpretation. *Language and Linguistics Compass*, 9(8), 311–327.

[CIT0171] Stephan, K. E., Penny, W. D., Moran, R. J., den Ouden, H. E., Daunizeau, J., & Friston, K. J. (2010). Ten simple rules for dynamic causal modeling. *Neuroimage*, 49(4), 3099–3109. 19914382 10.1016/j.neuroimage.2009.11.015 PMC2825373

[CIT0172] Strijkers, K., Bertrand, D., & Grainger, J. (2015). Seeing the same words differently: The time course of automaticity and top-down intention in reading. *Journal of Cognitive Neuroscience*, 27(8), 1542–1551. 25761003 10.1162/jocn_a_00797

[CIT0173] Swaab, T. Y., Ledoux, K., Camblin, C. C., & Boudewyn, M. A. (2012). Language-related ERP components. In S. J. Luck & E. S. Kappenman (Eds.), *Oxford library of psychology. The Oxford handbook of event-related potential components* (pp. 397–439). New York, NY: Oxford University Press.

[CIT0174] Szewczyk, J. M., & Schriefers, H. (2018). The N400 as an index of lexical preactivation and its implications for prediction in language comprehension. *Language, Cognition and Neuroscience*, 33(6), 665–686.

[CIT0175] Taylor, W. (1953). Cloze procedure: A new tool for measuring readability. *Journalism & Mass Communication Quarterly*, 30, 414–433.

[CIT0176] Taylor, J. S., Rastle, K., & Davis, M. H. (2013). Can cognitive models explain brain activation during word and pseudoword reading? A meta-analysis of 36 neuroimaging studies. *Psychological Bulletin*, 139(4), 766–791. 23046391 10.1037/a0030266

[CIT0177] Thornhill, D. E., & Van Petten, C. (2012). Lexical versus conceptual anticipation during sentence processing: Frontal positivity and N400 ERP components. *International Journal of Psychophysiology*, 83(3), 382–392. 22226800 10.1016/j.ijpsycho.2011.12.007

[CIT0178] van Berkum, J. J., Hagoort, P., & Brown, C. M. (1999). Semantic integration in sentences and discourse: Evidence from the N400. *Journal of Cognitive Neuroscience*, 11(6), 657–671. 10601747 10.1162/089892999563724

[CIT0179] Van Petten, C. (1993). A comparison of lexical and sentence-level context effects in event-related potentials. *Language and Cognitive Processes*, 8, 485–531.

[CIT0180] Van Petten, C., & Kutas, M. (1990). Interactions between sentence context and word frequency in event-related brain potentials. *Memory & Cognition*, 18, 380–393. 2381317 10.3758/bf03197127

[CIT0181] Van Petten, C., & Kutas, M. (1991). Influences of semantic and syntactic context on open and closed-class words. *Memory & Cognition*, 19, 95–112. 2017035 10.3758/bf03198500

[CIT0182] Van Petten, C., & Luka, B. J. (2012). Prediction during language comprehension: Benefits, costs, and ERP components. *International Journal of Psychophysiology*, 83(2), 176–190. 22019481 10.1016/j.ijpsycho.2011.09.015

[CIT0183] Varela, F., Lachaux, J., Rodriguez, E., & Martinerie, J. (2001). The brainweb: Phase synchronization and large-scale integration. *Nature Reviews Neuroscience*, 2(4), 229–239. 11283746 10.1038/35067550

[CIT0184] Vidal, J. R., Freyermuth, S., Jerbi, K., Hamamé, C. M., Ossandon, T., Bertrand, O., … Lachaux, J.-P. (2012). Long-distance amplitude correlations in the high gamma band reveal segregation and integration within the reading network. *The Journal of Neuroscience*, 32(19), 6421–6434. 22573665 10.1523/JNEUROSCI.4363-11.2012 PMC6621125

[CIT0185] Vignali, L., Himmelstoss, N. A., Hawelka, S., Richlan, F., & Hutzler, F. (2016). Oscillatory brain dynamics during sentence reading: A fixation-related spectral perturbation analysis. *Frontiers in Human Neuroscience*, 10, 191. 27199713 10.3389/fnhum.2016.00191 PMC4850157

[CIT0186] von Stein, A., Chiang, C., & König, P. (2000). Top-down processing mediated by interareal synchronization. *Proceedings of the National Academy of Sciences USA*, 97(26), 14748–14753. 10.1073/pnas.97.26.14748 PMC18990 11121074

[CIT0187] Wang, L., Hagoort, P., & Jensen, O. (2018a). Language prediction is reflected by coupling between frontal gamma and posterior alpha oscillations. *Journal of Cognitive Neuroscience*, 30(3), 432–447. 28949823 10.1162/jocn_a_01190

[CIT0188] Wang, L., Hagoort, P., & Jensen, O. (2018b). Gamma oscillatory activity related to language prediction. *Journal of Cognitive Neuroscience*, 30(8), 1075–1085. 29708821 10.1162/jocn_a_01275 PMC7618136

[CIT0189] Weiss, B., Knakker, B., & Vidnyánszky, Z. (2016). Visual processing during natural reading. *Scientific Reports*, 6, 26902. 27231193 10.1038/srep26902 PMC4882504

[CIT0190] Wenzel, M. A., Golenia, J.-E., & Blankertz, B. (2016). Classification of eye fixation related potentials for variable stimulus saliency. *Frontiers in Neuroscience*, 10, 23. 26912993 10.3389/fnins.2016.00023 PMC4753317

[CIT0191] Willems, R. M., Frank, S. L., Nijhof, A. D., Hagoort, P., & van den Bosch, A. (2016). Prediction during natural language comprehension. *Cerebral Cortex*, 26(6), 2506–2516. 25903464 10.1093/cercor/bhv075

[CIT0192] Wlotko, E. W., & Federmeier, K. D. (2012). Age-related changes in the impact of contextual strength on multiple aspects of sentence comprehension. *Psychophysiology*, 49(6), 770–785. 22469362 10.1111/j.1469-8986.2012.01366.x PMC4001119

[CIT0193] Wlotko, E. W., & Federmeier, K. D. (2015). Time for prediction? The effect of presentation rate on predictive sentence comprehension during word-by-word reading. *Cortex*, 68, 20–32. 25987437 10.1016/j.cortex.2015.03.014 PMC4832567

[CIT0194] Woldorff, M. G. (1993). Distortion of ERP averages due to overlap from temporally adjacent ERPs: Analysis and correction. *Psychophysiology*, 30(1), 98–119. 8416067 10.1111/j.1469-8986.1993.tb03209.x

[CIT0195] Woodhead, Z. V. J., Barnes, G. R., Penny, W., Moran, R., Teki, S., Price, C. J., & Leff, A. P. (2014). Reading Front to Back: MEG evidence for early feedback effects during word recognition. *Cerebral Cortex*, 24(3), 817–825. 23172772 10.1093/cercor/bhs365 PMC3920772

[CIT0196] Yan, S., Kuperberg, G. R., & Jaeger, F. (2017). Prediction (or not) during language processing. A commentary on Nieuwland et al. (2017) and Delong et al. (2005). *bioRxiv*. 10.1101/143750

[CIT0197] Yvert, G., Perrone-Bertolotti, M., Baciu, M., & David, O. (2012). Dynamic causal Modeling of Spatiotemporal integration of phonological and semantic processes: An electroencephalographic study. *The Journal of Neuroscience*, 32(12), 4297–4306. 22442091 10.1523/JNEUROSCI.6434-11.2012 PMC3948354

